# Laccase Immobilization on Tannic Acid–Coated Nylon Membrane for Dye Decolorization

**DOI:** 10.1021/acsomega.6c00521

**Published:** 2026-08-05

**Authors:** Claudia Sciacca, Nunzio Cardullo, Marcello Condorelli, Libera Vitiello, Andrea A. Scamporrino, Sabrina C. Carroccio, Vera Muccilli

**Affiliations:** † Department of Chemical Sciences, 9298 University of Catania, V. le A. Doria 6, 95125 Catania, Italy; ‡ Institute for Polymers, Composites and Biomaterials CNR, Via P. Gaifami 18, 95126 Catania, Italy

## Abstract

Laccase catalyzes the oxidation of a wide array of substrates, yet its industrial applications are hindered by its low thermal and chemical stability, poor reusability, and high production costs. Enzyme immobilization is a cost-effective strategy for overcoming these limitations, enhancing stability and operational efficiency. In this study, laccase from *Trametes versicolor* (LTV) was immobilized onto a nylon membrane coated with 3-aminopropyltriethoxysilane (APTES) and tannic acid (TA). A mathematical model was employed to optimize enzyme immobilization parameters (e.g., pH, LTV concentration, TA:APTES ratio, and reaction time), resulting in an enzyme loading of 31.9% w/w LTV and immobilization yield of 67.3%. The resulting biocatalytic system was characterized via ATR-FTIR, SEM–EDX, Raman, and fluorescence techniques, and the kinetics of free and immobilized LTV were analyzed. Immobilized LTV successfully oxidized 2,2′-azino-bis (3-ethylbenzothiazoline-6-sulfonic acid) across broad pH (2–9) and temperature (20 °C–70 °C) ranges. Compared to the free enzyme, immobilized LTV demonstrated improved storage stability and catalytic performance. Notably, immobilized LTV successfully decolorized methylene blue and methyl red dyes, validating the utility of this platform for environmental remediation applications.

## Graphical abstract

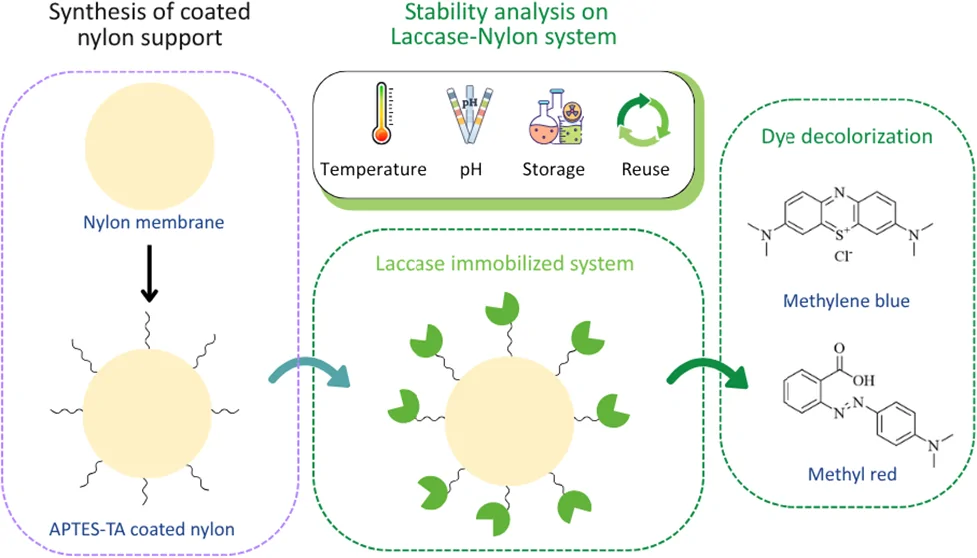

## Introduction

Laccase (benzenediol: oxygen oxidoreductase, EC 1.10.3.2) is a multicopper oxidase widely distributed across fungi, plants, bacteria, and certain insects.^1–3^ As a green and versatile biocatalyst, laccase oxidizes various phenolic and non-phenolic substrates,^2,4^ including lignin-related compounds^5^ and diverse environmental pollutants.^3,6,7^ These features make it attractive for a variety of industrial sectors, including food processing,^8^ pulp and paper,^9^ textiles,^10^ and pharmaceuticals.^11^ Fungal laccases are of particular interest owing to their high redox potential, which typically ranges from 470 to 810 mV.^12^ Among these, laccase from the mushroom *Trametes versicolor* (LTV) exhibits one of the highest reported redox potentials (∼800 mV), resulting in robust catalytic activity against a variety of organic substrates.^13,14^ However, industrial applications of laccase are hindered by its low chemical and thermal stability, poor reusability, and high production costs.^15^ Enzyme immobilization effectively overcomes these drawbacks, enhancing stability across broad pH and temperature ranges and facilitating recovery for reuse.^12,16,17^

To enhance the catalytic performance and thermal stability of laccase, recent studies have investigated its immobilization on different supports, including poly­(glycidyl methacrylate)-based microspheres,^18^ polystyrene microspheres functionalized with diazonium groups,^19^ polyacrylonitrile–polyether sulfone material,^20^ 3D-printed polylactic acid filaments,^21^ chitosan-functionalized halloysite nanotubes,^22^ functionalized multiwalled carbon nanotubes,^23^ titania nanoparticles and titania-functionalized membranes,^24^ functionalized carbon–polysulfone membranes,^25^ nylon-Fe^3+^ composite nanofibrous membranes,^26^ electrospun polycaprone–polyehyleneimine fiber membranes,^27^ polylactic acid electrospun fibers,^28^ ground silk fibroin nanofibers,^29^ natural silica,^30^ and Fe_3_O_4_–SiO_2_ chitosan nanoparticles.^31^ Among these supports, nylon membranes are an attractive option owing to their mechanical strength, flexibility, chemical resistance, and ease of surface modification.^32,33^

To improve the surface functionality of nylon membranes, this study explores a bioinspired coating based on tannic acid (TA). TA, which is a mixture primarily composed of various galloylated glucopyranoses and gallotannins,^34,35^ is a widely available, cost-effective, and environmentally friendly product. Figure 1 illustrates the characteristic *meta*-depsidic digallolyl group of TA. In solution, these *meta*-depside bonds rapidly equilibrate into *para*-depside bonds, which reduces the number of pyrogallol units containing adjacent free phenolic hydroxyl groups capable of being converted into *ortho*-quinones. Tannin-based extracts and TA exhibit universal adhesiveness through intermolecular interactions with various nucleophiles (e.g., amines, amide bonds, and thiols), displaying strong metal-chelating properties and readily forming supramolecular conjugations with biomacromolecules and polymers. Consequently, TA can be exploited as a cross-linker, enabling the formation of versatile polymeric networks that facilitate adherence to various surfaces.^36,37^ However, the oxidation of pyrogallol moieties to quinones weakens the stability of TA coatings, degrading surface adhesion over time.^38,39^ To address this stability issue, this study codeposits TA with 3-aminopropyltriethoxysilane (APTES) on a nylon membrane to construct a homogeneous, stable coating that provides an ideal adhesive surface for enzyme immobilization (Figure 1).^39–41^ Herein, TA is represented by the structural features common to its constituents that participate in the proposed chemical transformations.

**Figure 1:**
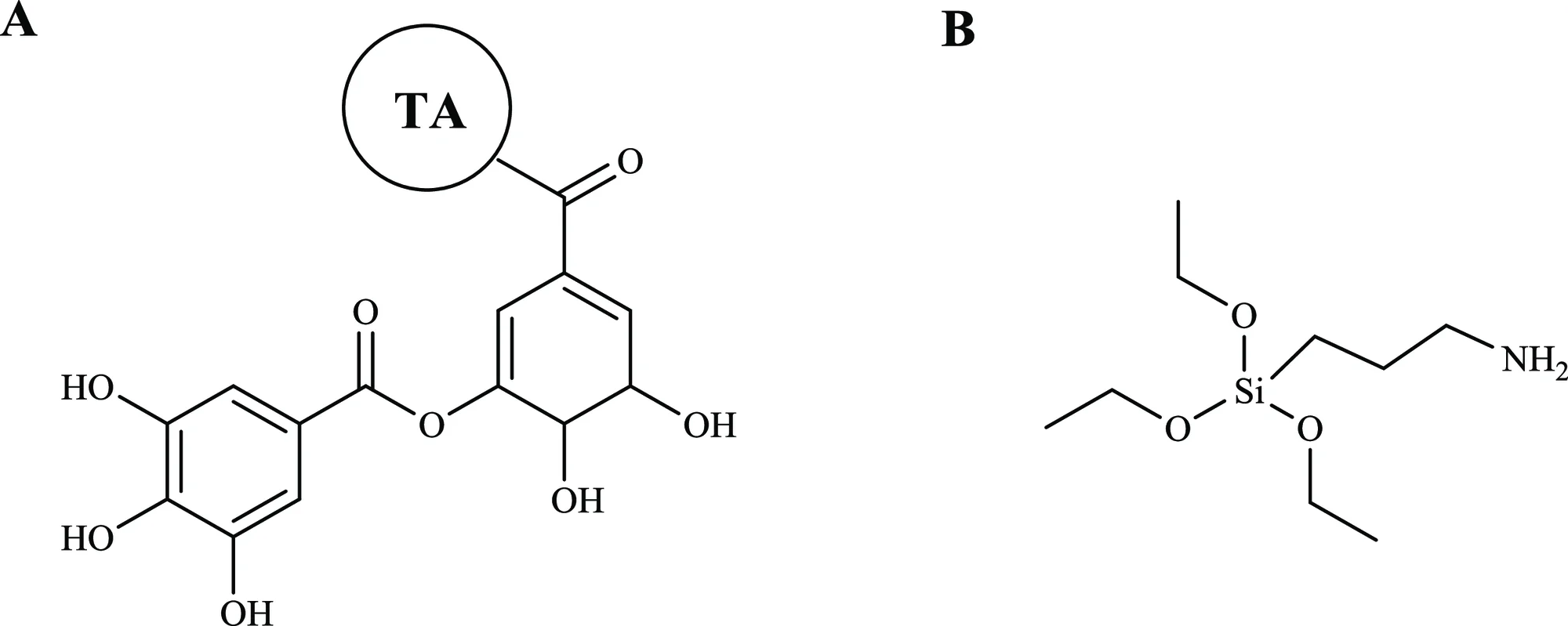
**A)** Gallotannin *meta*-depsidic digalloyl group, and B) 3-aminopropyltriethoxysilane (APTES).

As illustrated in Figure 2, this study presents an advanced support that anchors laccase through the chemical modification of TA functionalities. This novel platform expands the feasibility of laccase-based biocatalysis in demanding environments, such as pollutant degradation in wastewater, thereby contributing to ecofriendly industrial processing.

**Figure 2:**
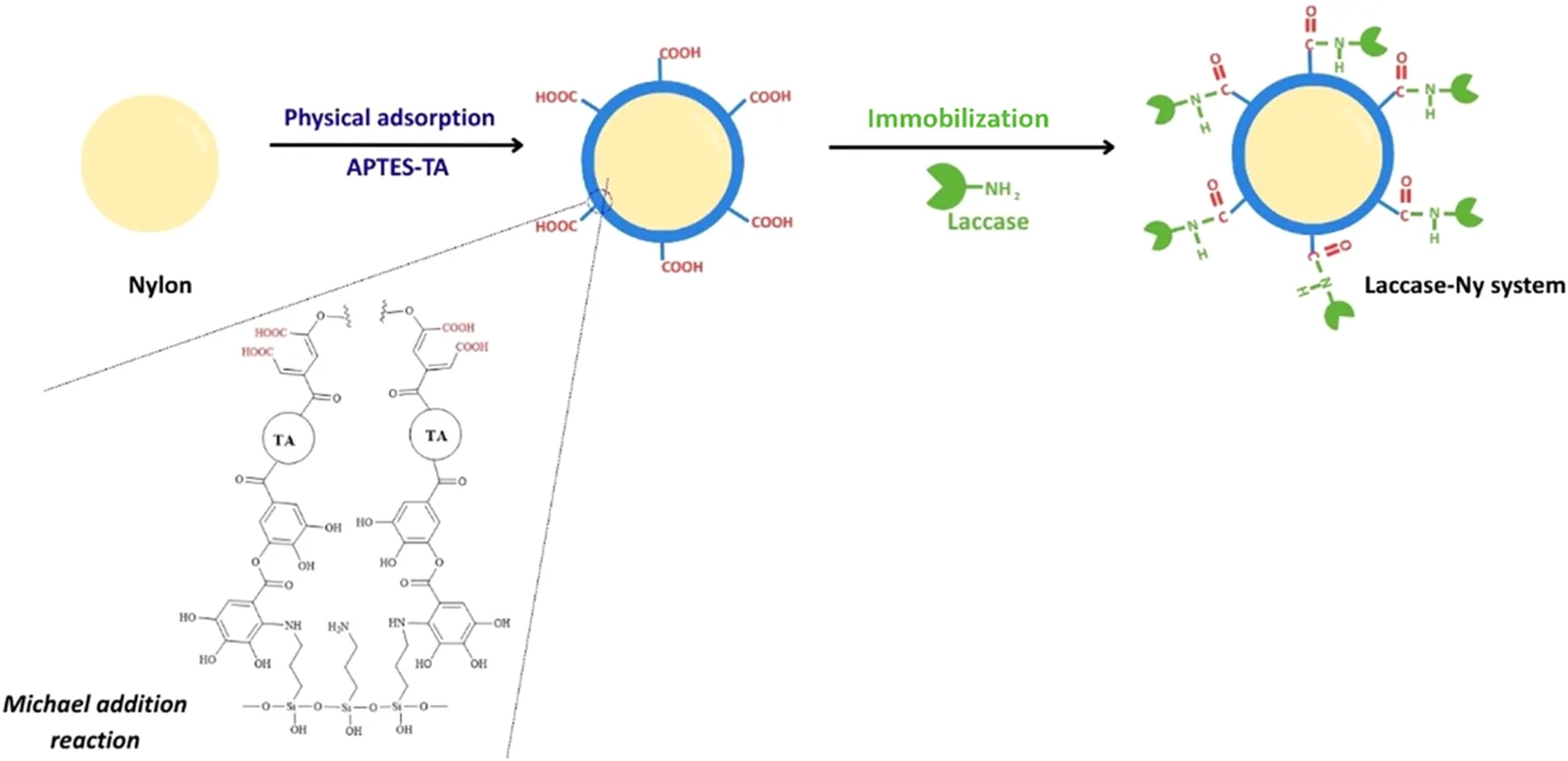
Schematic representation of the procedure for the immobilization of laccase from *Trametes versicolor* (LTV) onto nylon membrane (Laccase-Ny system).

## Experimental Section

### Chemicals and Reagents

The commercial fungal laccase from *Trametes versicolor* (EC 1.10.3.2/CAS N: 80498-15-3) used in this work was purchased from Sigma-Aldrich (USA) and the batch employed in this study, BCCK0654, exhibited an activity greater than 0.5 U/mg. 3-Aminopropyltriethoxysilane (APTES), NaIO_4_, Coomassie Brilliant Blue, and sodium phosphate dibasic heptahydrate (Na_2_HPO_4_) were obtained from Sigma-Aldrich with the highest available purity grade. 2,2′-Azino-bis­(3-ethylbenzothiazoline-6-sulfonic acid) (ABTS), *N,N*′-dicyclohexylcarbodiimide (DCC) and 8-anilino-1-naphthalenesulfonic acid (ANS) were purchased from Tokyo Chemical Industry (TCI). Tannic acid (TA) was obtained from Thermo Fisher Scientific. Sodium acetate, potassium dihydrogen phosphate (KH_2_PO_4_), methylene blue (MB) and methyl red (MR) were purchased from Carlo Erba. Acetic acid and hexane-1,6-diamine were supplied by Fluka. Nylon membranes (pore size 0.22 μm) were purchased from FUS North America. The nylon membranes were cut into discs with 0.8 cm diameter, and used for synthesis as described below. All other chemicals and reagents used were of the highest available purity.

### Experimental Design for Optimization of Laccase Immobilization

To determine the optimal conditions for the immobilization of LTV onto APTES - TA coated nylon membrane response surface methodology (RSM) was applied using a Box-Behnken design (BBD). The experimental design evaluated the influence of four independent variables (*X*_1_ - *X*_4_) – buffer pH, LTV concentration (mg/mL), TA:APTES ratio, and immobilization reaction time – as reported in Table S1. The design matrix, consisting of 27 experimental runs, was generated with JMP statistical software (SAS Institute S.r.l, Milano, Italy), following the equation:

1 $$N=2k\left(k-1\right){+C}_{0}$$

where *k* is the number of factors, and C_0_ is the number of the central points. Each factor was investigated at three distinct levels designed as −1 for the low level, 0 for the medium level, and +1 for the high level. The main response variable measured was the percentage of laccase immobilized on the nylon membrane, quantified by the Bradford protein assay. The experimental data were fitted to a second-order polynomial regression model form:

2 $$Y=B_{0+}\sum_{i=1}^{k}B_{i}X_{i}+\sum_{i=1}^{k}B_{\mathrm{ii}}X^{2}+\sum_{i=1}^{k}B_{\mathrm{ij}}X_{i}X_{\mathrm{ij}}+E$$

where *Y* represents the predicted response, *X* represents the independent variable, *B*_0_ is the intercept and *B*_*i*_, *B*_*ii*_, and *B*_*ij*_ are respectively, the linear, quadratic, and interactive regression coefficients. Analysis of variance (ANOVA) was employed to estimate the regression coefficients, and statistical significance was determined using Tukey’s test with a *p-*value <0.05. Three-dimensional response surface plots were generated to visually explore the interaction effects among each variable and to identify factor combinations that maximize immobilization efficiency. Model performance was analyzed through the calculation of the coefficient of determination (*R* ^*2*^), the *F*-test values, and the lack-of-fit test. The optimized conditions were subsequently validated in triplicate to confirm reproducibility and effectiveness of the laccase immobilization process.

### Preparation of APTES - TA Coated Nylon Membrane

The nylon membrane discs (0.8 cm; 2.5 ± 0.2 mg) were soaked in ethanol for 6 h to remove any impurities from the membrane pores. Each disc was then immersed in deionized water for 1 h to remove excess ethanol from the membrane pores^40^ and finally stored in pure water until further use. A sodium periodate solution (2 mg/mL; 0.5 mL) prepared in 50 mM Tris-HCl buffer (pH 8.5),^40^ was mixed with TA (2 mg/mL in Tris-HCl; 1 mL) and 2.14 M APTES ethanol solution (0.15 mL). The prewetted nylon disc was immediately immersed in the reaction solution, and the mixture was shaken at 60 °C and 200 rpm for 1 h. After treatment, the membrane was removed and rinsed vigorously with pure water to remove any unbound chemicals on its surface and then dried for further use.^40^

### Activation of APTES-TA Coated Nylon Membrane

The resulting coated nylon membrane (3.2 ± 0.2 mg) was reacted with hexane-1,6-diamine (4.8 mg; 1% w/v) and DCC (5.3 mg; 1% w/v) previously solubilized in CH_2_Cl_2_ (0.5 mL). After shaking at room temperature, at 200 rpm, for 4h, the membrane was washed in the following order: acetone (0.5 mL), dichloromethane (0.5 mL), ice-cold water (0.5 mL), and then dried for the immobilization step.^42,43^

### Immobilization of Laccase onto Nylon-APTES-TA Membrane (Laccase-Ny)

LTV (0.3 mg/mL in 50 mM acetate buffer, pH 5) was added to the activated APTES-TA coated nylon membrane (3.6 ± 0.2 mg) and incubated on a shaker (200 rpm) at room temperature for 18h and 33 min. Finally, the Laccase-Ny system was washed with acetate buffer and dried. In addition, a laccase solution was added to pristine nylon (2.5 ± 0.2 mg) and incubated for the same time to assess whether the enzyme could be retained through non-covalent interactions (control system).

### Characterization of Nylon Membrane with and without Immobilized Laccase

The surface of the coated support, the Laccase-Ny system, and the control system (ATR-FTIR, PerkinElmer Frontier Spotlight 400). All the spectra were recorded in the 400–4000 cm^–1^ range, with a spectral resolution of 2 cm^–1^, and 12 scans were acquired and averaged to obtain a single spectrum, thereby improving the signal-to-noise ratio. The surface morphology and elemental composition of the nylon membrane with immobilized laccase were examined by Scanning Electron Microscopy (SEM) on a Phenom Desktop G5 (PhenomWorld, Eindhoven, Netherlands) equipped with an Energy-Dispersive X-ray (EDX) detector. Before imaging, samples have been fixed onto aluminum stubs and coated with a thin layer of gold (<10 nm) to improve electrical conductivity. Elemental mapping and spectra were collected and analyzed using Phenom Element Identification software (version 3.8.4.0, Phenom-World Pro-Suite 2.9.0.0 bundle, Eindhoven, The Netherlands).

### Quantification of Immobilized Laccase onto APTES - TA Coated Nylon Membrane

The total amount of LTV (% w/w) effectively immobilized on the coated nylon membrane was determined by the Bradford protein assay using the standard addition method.^28^ In a 96-well plate, each standard solution of laccase (10–75 μg/mL; 60 μL) was added from the least to the most concentrated, then 0.1 M acetate buffer, pH 4.5 (60 μL), and the Laccase-Ny membrane (4.2 ± 0.3 mg) was added to each well. Finally, Bradford reagent (120 μL) was added. The plate was shaken for 1 min, and absorbance was measured at 620 nm after 20 min. In this experiment, APTES - TA coated nylon membrane (3.2 ± 0.2 mg) was assayed under the same conditions and used as a blank. The same procedure was repeated for the control system, using pristine nylon (2.5 ± 0.2 mg) as a blank. All assays were performed in triplicate.

### Laccase Activity Assay

LTV activity was measured by monitoring ABTS oxidation at 420 nm using a JASCO V-670 spectrophotometer. Briefly, the Laccase-Ny system (4.2 ± 0.3 mg) was mixed with 0.1 M acetate buffer (pH 4.5, 1.9 mL), 0.1 mL of water, and 0.05 mM ABTS solution (1.0 mL). Experiments without the Laccase-Ny system were performed as blanks. Free LTV (0.3 mg/mL; 0.1 mL) was employed as a positive reference. LTV activity was subsequently determined employing the molar extinction coefficient of 36,000 M^–1^ cm^–1^.^44^ One unit (U) of LTV activity corresponds to the oxidation of 1 μmol ABTS per minute under the specified assay conditions. All the assays were performed in triplicate. Data were processed according to the following formula:^45^

3 $$immobilized\;enzyme\;activity\;\left(U/g\right)=\frac{\Delta A\times V_{t}{\times 10}^{6}}{\Delta t\times \epsilon \times W}$$

where Δ*A* is the change in absorbance at 420 nm, *V_t_* is the volume of the reaction system, ε is the molar extinction coefficient of ABTS, Δ*t* is the reaction time (min), and *W* is the weight of the Laccase-Ny system or of the laccase employed.

The immobilization efficiency was expressed in terms of the immobilization yield according to the following equation:^15^

4 $$\mathrm{immobilization}\;\mathrm{yield}\;\left(\%\right)=\frac{L_{i}-L_{f}}{L_{i}}\times 100\%$$

Where *L_i_* is the laccase activity before immobilization, and *L*_*f*_ is the Laccase-Ny System activity.

### Laccase Reusability Test

The reusability of the Laccase-Ny system (4.2 ± 0.3 mg) was monitored over 15 cycles using ABTS as the substrate. After each cycle, the immobilized enzyme support was recovered from the reaction mixture and washed with 0.1 M acetate buffer (pH 4.5). The assays were performed in triplicate, and LTV activity was measured as previously described to assess any loss in activity and potential reusability. The initial activity was set as 100%, and the relative activity was calculated as reported in the following equation:^44,46^

5 $$relative\;activity\;\left(\%\right)=\frac{measured\;enzyme\;activity}{initial\;enzyme\;activity}\times 100$$

### Effect of pH, Temperature, and Time on Stability

Free and immobilized LTV were subjected to stability studies. Thermal stability was assessed by incubating the samples in 50 mM acetate buffer (pH 4.5), at 20, 25, 30, 40, 50, 60, and 70 °C for 24 h, while storage stability was assessed at pH 4.5, at 4 °C for 30 days.^46^ The effect of pH on LTV activity was evaluated by incubating samples at 25 °C for 24 h at pH: 2.0, 3.0, 4.0, 4.5, 5.0, 6.0, 7.0, 8.0 9.0.^47^ The buffers used were 50 mM HCl/KCl (pH 2), 50 mM acetate buffer (pH 3–5), 50 mM phosphate buffer (pH 6–7), and 50 mM tris-HCl (pH 8–9). According to eq 5, stability was estimated as the residual LTV activity relative to the initial enzyme activity. All assays were performed in triplicate.

### Kinetic Analysis of Free and Immobilized Laccase

Kinetic parameters of the free and immobilized LTV were determined by measuring the reaction of LTV with the substrate ABTS. Various ABTS concentrations were used, ranging from 0.03 to 3.0 mM for the free enzyme and from 0.03 to 5.8 mM for the immobilized laccase^48^ under standard reaction conditions (25 °C, 0.1 M acetate buffer, pH 4.5). The experiments were performed in triplicate. The Michaelis–Menten constant (*K*_*m*_) is the substrate concentration at which the reaction rate reaches half of its maximum value (*V*_max_).^46^ The kinetic parameters were obtained using a Lineweaver–Burk plot based on the following equation:

6 $$\frac{1}{V}=\frac{K_{m}}{V_{\max}}\times \frac{1}{\left[S\right]}+\frac{1}{V_{\max}}$$

where *V*_max_ is the reaction velocity (mM/min) and [*S*] is the substrate concentration (mM).

### Raman Measurements

Protein spectra before and after deposition on Nylon were examined by Raman spectroscopy, using a Nd:YAG laser (532 nm) on a WITec α 300 (Wissenschaftliche Instrumente and Technologie GmbH, Ulm, Germany) confocal Raman apparatus. The laser power was set at 0.5 mW to avoid potential alteration of the samples. Spectra were collected by a 100× objective, with an integration time of 10 s per spectrum and 10 accumulations, and were analyzed with OriginPro (version 9.5, OriginLab, Northampton, MA, USA).

### Fluorescence Analysis

Intrinsic fluorescence assays were performed in a 96-well microplate with a total volume of 200 μL per well. Fluorescence spectra of free and immobilized LTV support (4.2 ± 0.3 mg) were recorded at 25 °C with an excitation wavelength of 280 nm and monitored over the range of 300–500 nm, with a final LTV concentration of 0.03 mg/mL in acetate buffer (0.1 M, pH 4.5). Thermal fluorescence studies of free and immobilized LTV were conducted following the same parameters over a temperature range of 25–45 °C, corresponding to the maximal operating temperature achievable by the instrument. Extrinsic fluorescence measurements were performed using 8-anilino-1-naphthalene-sulfonic acid (ANS) as the fluorescent probe. The assay was carried out in a 96-well microplate with a total volume of 200 μL per well. Fluorescence spectra were recorded at 25 °C in 0.1 M acetate buffer (pH 4.5) containing the free or immobilized laccase and ANS at final concentrations of 1 μM and 30 μM, respectively. ANS fluorescence emission was measured over the range of 400–600 nm with an excitation wavelength of 350 nm.

### Decolorization Measurements

A mixture of methylene blue (MB) and methyl red (MR) with a concentration of 0.03 mg/mL (of each dye) in 0.1 M acetate buffer (pH 4.5) was incubated with Laccase-Ny system (8.4 ± 0.2 mg) at 25 °C and 200 rpm for 24 h. Aliquots (150 μL) were collected at predetermined time intervals (1, 2, 4, 6, 8, 24 h). LTV (0.3 mg/mL) was used in a comparative experiment, and pristine nylon was subjected to an identical reaction to verify any adsorption phenomena that could cause decolorization. Experiments were performed in triplicate and expressed as mean ± standard deviation (SD; *n* = 3). The residual dyes content in the experiments was determined by high-performance liquid chromatography (HPLC-UV), acquiring chromatograms at 665 nm (for MB) and 530 nm (for MR). The analyses were carried out on a C-18 reversed-phase column (5 μm, 4.60 mm × 250 mm, Phenomenex, Castel Maggiore, BO, Italy) kept at 25 °C, and using a mobile phase consisting of (A) water with 0.1%(v/v) of formic acid and (B) acetonitrile with 0.1%(v/v) of formic acid. The solvent gradient was set as follows: 0–13 min, 30–95% B; 13–14 min, 95% B; 14–20 min, 95–30% B, at 1 mL/min. The percentage of dye decolorization was quantified using the following equation:

7 $$\mathrm{decolorization}\;\left(\%\right)=\frac{C_{0}-C_{f}}{C_{0}}\times 100$$

Where, *C*_0_ is the absorbance corresponding to the dye initial concentration; *C*_*f*_ is the absorbance corresponding to dye concentration at the selected time interval.

## Results and Discussion

### Development of Laccase-Nylon System

As detailed in Figure 3, a nylon membrane modified with a codeposited APTES–TA coating was developed to immobilize LTV. The APTES–TA layer was synthesized for the first time on a nylon membrane via a one-pot procedure adapted from the literature.^40^ APTES is an organosilane compound commonly used for surface functionalization. Briefly, its alkyloxy groups hydrolyze into highly reactive silanol-containing species, which undergo self-condensation to form a polymerized siloxane network.^49,50^ Simultaneously, the amine (−NH_2_) groups of APTES cross-link with the TA network physically adsorbed on the nylon membrane. Given that appropriate oxidants facilitate the conversion of TA pyrogallol groups into quinone structures to accelerate the deposition of polyphenolic compounds,^51^ NaIO_4_ was employed to generate these quinones and to drive the oxidative cleavage of the TA skeleton, yielding carboxylic acid (−COOH) groups.^52^ The resulting quinone functional groups can react with the amine (−NH_2_) groups of APTES via two pathways: direct 1,2 addition to the carbonyl group (Schiff base formation) or 1,4 Michael addition to the conjugated quinone system.^40^ Under the reported conditions, the reaction likely proceeds via 1,4 Michael addition between APTES and the oxidized form of TA.

**Figure 3:**
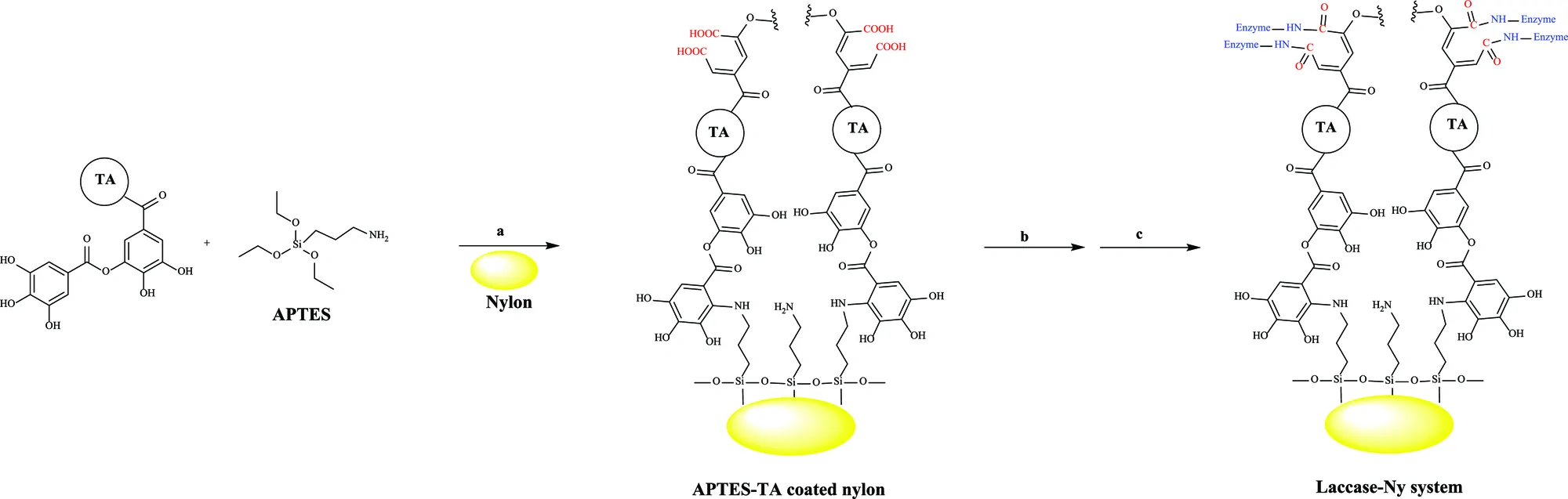
Schematic representation of the synthesis of the Laccase-Ny system. a) Coating step: 50 mM Tris HCl buffer, pH 8.5; sodium periodate, 60 °C, 1h, 200 rpm. b) Activation step: DCC, hexane-1,6-diamine, CH_2_Cl_2_, 25 °C, 4h, 200 rpm; c) Immobilization step: laccase from *Trametes versicolor* in buffer solution.

In the second step, the −COOH groups generated during TA oxidation might react with the amine groups of LTV amino acid residues to form stable amide bonds. To achieve this purpose, the −COOH groups of TA were activated using DCC, adopting conditions previously reported for small molecules such as terephthalic acid.^42,43^ Subsequently, the nylon membrane coated with activated APTES–TA was reacted with LTV. To maximize enzyme immobilization efficiency, the reaction conditions were optimized using a Design of Experiments (DoE) approach, as detailed below. Notably, this study reports laccase immobilization via amide bonds for the first time, contrasting with previous studies that have primarily relied on Schiff base formation or Michael additions.^30,31,39,53–56^

### Optimization of Immobilization Conditions via Response Surface Methodology (RSM)

To optimize the factors affecting LTV immobilization on coated nylon membrane, an initial set of single-factor experiments was performed to define the parameter ranges for subsequent DoE runs. The factors evaluated included pH (*x*_1_), enzyme concentration (LTV; *x*_2_), TA:APTES ratio (v/v; *x*_3_), and reaction time (*x*_4_). These preliminary experiments were performed by varying a single parameter, while maintaining all others at central values (pH 5; LTV concentration 0.9 mg/mL; TA:APTES ratio 4:1; reaction time 24 h; see Table S1). The effective amount of immobilized enzyme (% w/w LTV) was determined using the Bradford protein assay. As shown in Table S2, the amount of immobilized enzyme increased from 0.23 to 14.01% as the pH rose to 5 but decreased from pH 6.5 to 8.5, indicating an optimal pH range of 3–5. Increasing the initial enzyme concentration beyond 0.6 mg/mL (20.3% w/w LTV) did not improve the functionalization of the modified nylon membrane. Moreover, the amount of immobilized LTV improved from 2.88 to 18.40% as the TA:APTES ratio increased from 1:1 to 8:1. Regarding the reaction time, the amount of immobilized LTV reached a maximum of 14.01% at 24 h and then decreased, indicating an optimal time window of 8–24 h.

Following these preliminary findings, parametric optimization was conducted using a Box–Behnken design (BBD) approach to evaluate the influence of the four variables (*x*_1_–*x*_4_) on the dependent response (*Y* = LTV % w/w, Table S3). The DoE matrix generated 27 experimental runs (Table S4). Analysis of variance using least-squares regression (Table 1) indicated that all linear coefficients were significant (*p* < 0.05), with the exception of the TA:APTES ratio (*p* = 0.1631). Among the investigated variables, reaction time exerted the greatest influence on response *Y*, as indicated by its high *F*-value (194.76) and low *p*-value (<0.0001). Furthermore, all quadratic terms and the interaction terms *x*_1_ *x*_2_, *x*_1_ *x*_3_, and *x*_3_ *x*_4_ were significant (*p* < 0.0001, < 0.0001, and 0.0045, respectively). Overall, the model demonstrates strong predictive capability, as indicated by its high *F*-value (69.08) and low *p* values (<0.0001). Furthermore, the lack-of-fit test was not significant (*p* > 0.05), confirming the model’s suitability for parameter optimization. A high coefficient of determination of 0.9908 close to one verified a strong correlation between the measured and predicted data. Finally, a coefficient of variation of 4.22% indicated the high reliability of the experimental data, underscoring that all measurements performed during the DoE runs were consistent.^57^ The final predictive equations considered only the significant terms:

$$Y\left(\mathrm{LTV}\%\;w/w\right)=51.37676-25.1020x_{1}-11.6160x_{2}+2.4118x_{4}+3.0835x_{1}^{2}+56.2394x_{2}^{2}+0.3895x_{3}^{2}‐0.0551x_{4}^{2}-12.7272x_{1}x_{2}+1.9357x_{1}x_{3}-2.6322x_{2}x_{3}-0.0987x_{3}x_{4}$$

**Table 1:** Results of ANOVA from the BBD Model for the % Amount of LTV (Y_1_)

	sum of squares		*p*-value
variable	*Y*_1_	*Y*_1_	*Y*_1_
model	1024.87	69.08*F*-value	<0.0001^a^
*x_1_*	150.78	142.28	<0.0001^a^
*x_2_*	21.59	20.38	0.0015^a^
*x_3_*	2.44	2.30	0.1631
*x_4_*	206.39	194.76	<0.0001^a^
x_1_*x_2_	68.49	64.63	<0.0001^a^
x_1_*x_3_	180.29	170.13	<0.0001^a^
x_1_*x_4_	2.74	2.58	0.1424
x_2_*x_3_	9.14	8.63	0.0166
x_2_*x_4_	0.22	0.20	0.6619
x_3_*x_4_	14.97	14.12	0.0045^a^
x_1_*x_1_	47.76	45.06	<0.0001^a^
x_2_*x_2_	111.67	105.38	<0.0001^a^
x_3_*x_3_	32.34	30.52	0.0004^a^
x_4_*x_4_	50.60	47.75	<0.0001^a^
lack of fit	2.78	0.33	0.872
*R* ^2^	0.9908		
*R* ^2^(Adj.)	0.9764		

a Values statically significant at *p* < 0.05.

Response surface plots were generated to visualize the interactive effects of the various parameters governing LTV immobilization onto the modified nylon membrane (Figure 4A–F). As shown in Figure 4A–C, higher buffer pH values corresponded to greater LTV immobilization (% w/w), which was statistically supported by the significant quadratic term for pH (*p* < 0.0001). Additionally, increasing the reaction time improved enzyme loading (Figure 4C,E,F). This pattern is consistent with the results reported by Latif et al.,^58^ who observed a similar trend when immobilizing laccase on Ba-alginate beads. Furthermore, the amount of immobilized enzyme increased as the initial LTV concentration decreased from 0.9 to 0.3 mg/mL. This trend was similarly noted by Rybarczyk et al.^21^ when immobilizing LTV on polylactide filaments. The highly significant *x*_1_ *x*_2_ (*p* < 0.0001) term confirmed a strong relationship between pH and LTV concentration at a constant time and TA:APTES ratio. By contrast, the model revealed no significant relationship between pH and reaction time at a constant concentration and TA:APTES ratio (*p* = 0.1424) or between the enzyme concentration and reaction time at a constant pH and TA:APTES ratio (*p* = 0.6619).

**Figure 4:**
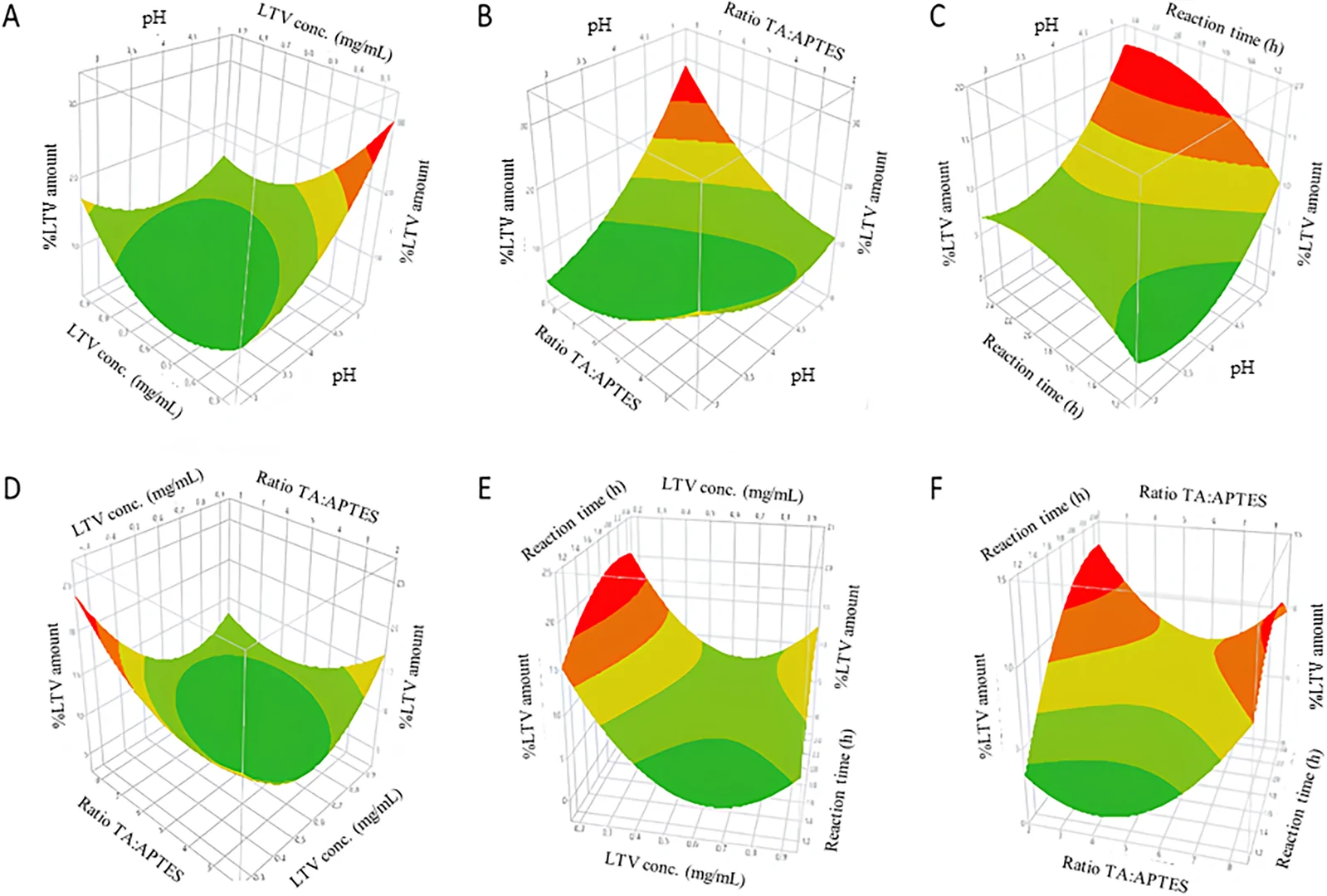
3-D surface plots for Laccase-Ny system as a function of: pH/laccase (LTV) concentration (A), pH/ratio TA:APTES (B), pH/reaction time (C), laccase (LTV) concentration/ratio TA:APTES (D), laccase (LTV) concentration/reaction time (E) and reaction time/ratio TA:APTES (F).

Finally, the optimal conditions for LTV immobilization on the modified nylon membrane were determined to be pH 5, an enzyme concentration of 0.3 mg/mL, a TA:APTES ratio of 8:1, and a reaction time of 18 h and 33 min. This optimal combination was experimentally validated in triplicate immobilization reactions, and the experimental data closely matched the predicted values, with no significant differences (Table S5; *p* < 0.05). The optimized conditions enabled an enzyme loading of 31.9% w/w LTV. The activity of the resulting Laccase–Ny system was evaluated using the ABTS method, revealing an immobilization yield of 67.3%. This value is comparable to that reported by Jasni et al.^26^ on nylon-6,6/Fe_3_O_4_ composite nanofibrous membranes and by Fatarella et al.^59^ on spacer-arm functionalized nylon after acidic hydrolysis. Notably, the immobilization yield was higher than that obtained by Khanam et al.^30^ on silica coated with TA–APTES. Furthermore, to assess whether the nylon membrane could bind LTV through multiple noncovalent interactions without surface functionalization, a pristine nylon membrane was reacted with LTV under optimal conditions (e.g., enzyme concentration, pH, and reaction time). The Bradford assay revealed no detectable enzyme loading.

### Characterization of Synthesized Support

The modified nylon membrane and Laccase–Ny system were analyzed via attenuated total reflection Fourier-transform infrared spectroscopy (ATR-FTIR), as shown in Figure 5. The ATR-FTIR spectrum of the modified nylon membrane (red spectrum) exhibited a broad absorption band at 3238 cm^–1^, attributed to the O–H stretching vibrations of TA. Distinctive signals at 1039 and 744 cm^–1^ corresponded to the Si–O–Si stretching vibrations of the condensed APTES structure. Notably, no absorption was detected at 1705 cm^–1^, typically assigned to the C=O stretching of the quinone form of oxidized TA. This signal was likely superimposed on the signal at 1685 cm^–1^, attributed to the carboxylic group (−COOH). No signal attributable to the imine bond (C=N) was detected, as imine functional groups formed via 1,2-additions would rapidly undergo hydrolytic cleavage back to quinone carbonyls under the reported reaction conditions. These observations suggest that the APTES–TA layer may form through a Michael-type addition. By contrast, the ATR-FTIR spectrum of the Laccase–Ny system (blue spectrum) exhibited some features confirming enzyme binding. A broad band at 3299 cm^–1^ was assigned to N–H stretching, while the signal at 3084 cm^–1^ corresponded to overtone bending vibrations. The peak at 1725 cm^–1^, attributable to the C=O stretching of carboxyl derivatives, was consistent with the newly formed amide bond. The peaks at 1538 and 690 cm^–1^ were associated with in-plane and out-of-plane N–H bending, respectively. Furthermore, the signal at 1275 cm^–1^ was attributed to C–N stretching vibrations. The ATR-FTIR spectrum of pristine nylon treated with LTV (control) was identical to that of the pristine nylon membrane (Figure S2), demonstrating that the enzyme did not bind or physically adsorb onto the nylon membrane in the absence of surface modification.

**Figure 5:**
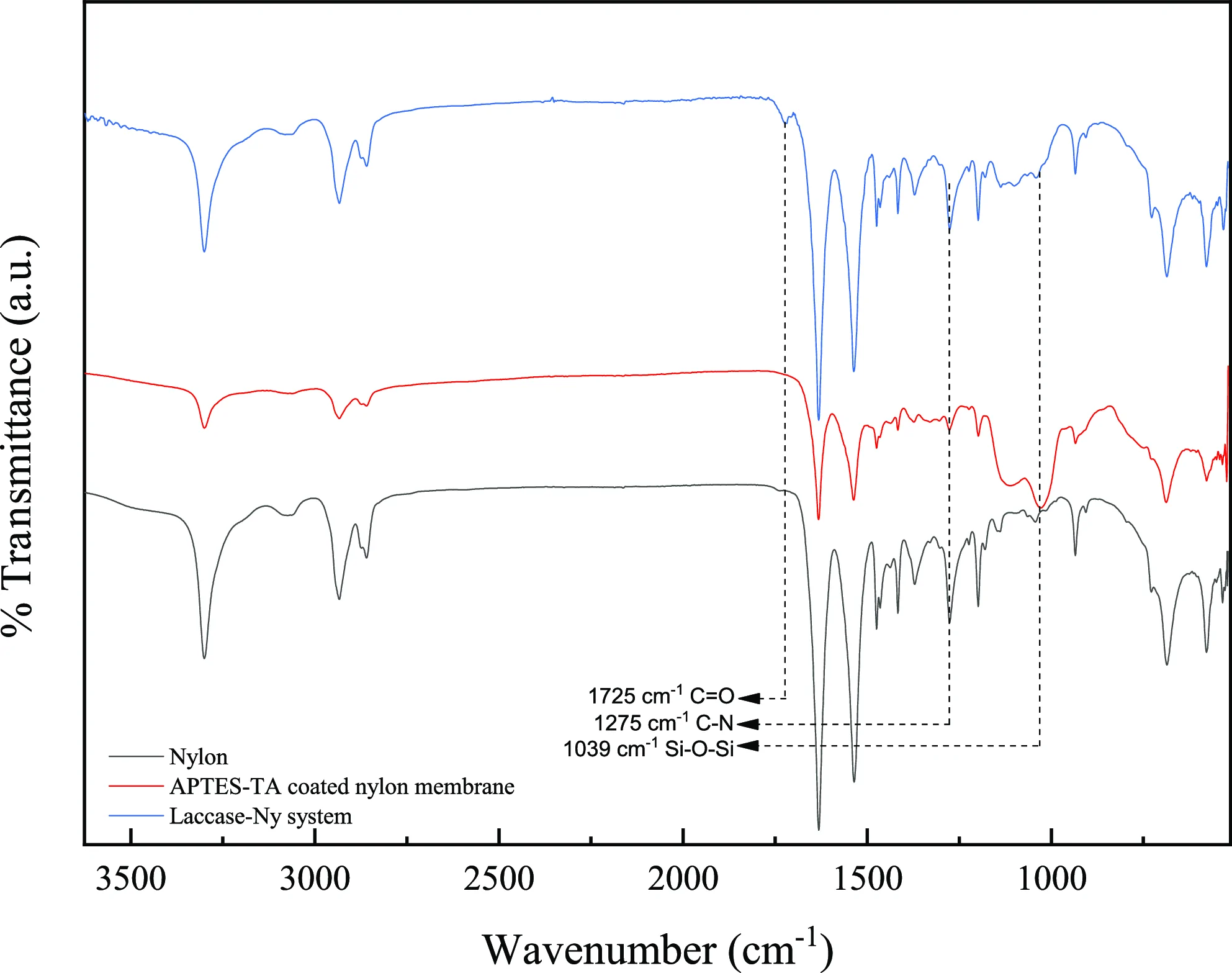
ATR-FTIR spectrum of nylon membrane (black), APTES-TA coated nylon membrane (red), and Laccase-Ny system (blue).

A SEM image of the Laccase–Ny system is presented in Figure 6A, revealing an irregular surface morphology with distinct silicon spheres, indicating successful surface functionalization. Elemental mapping via EDX revealed the presence of sulfur signals, confirming LTV immobilization (Figure 6B,C).

**Figure 6:**
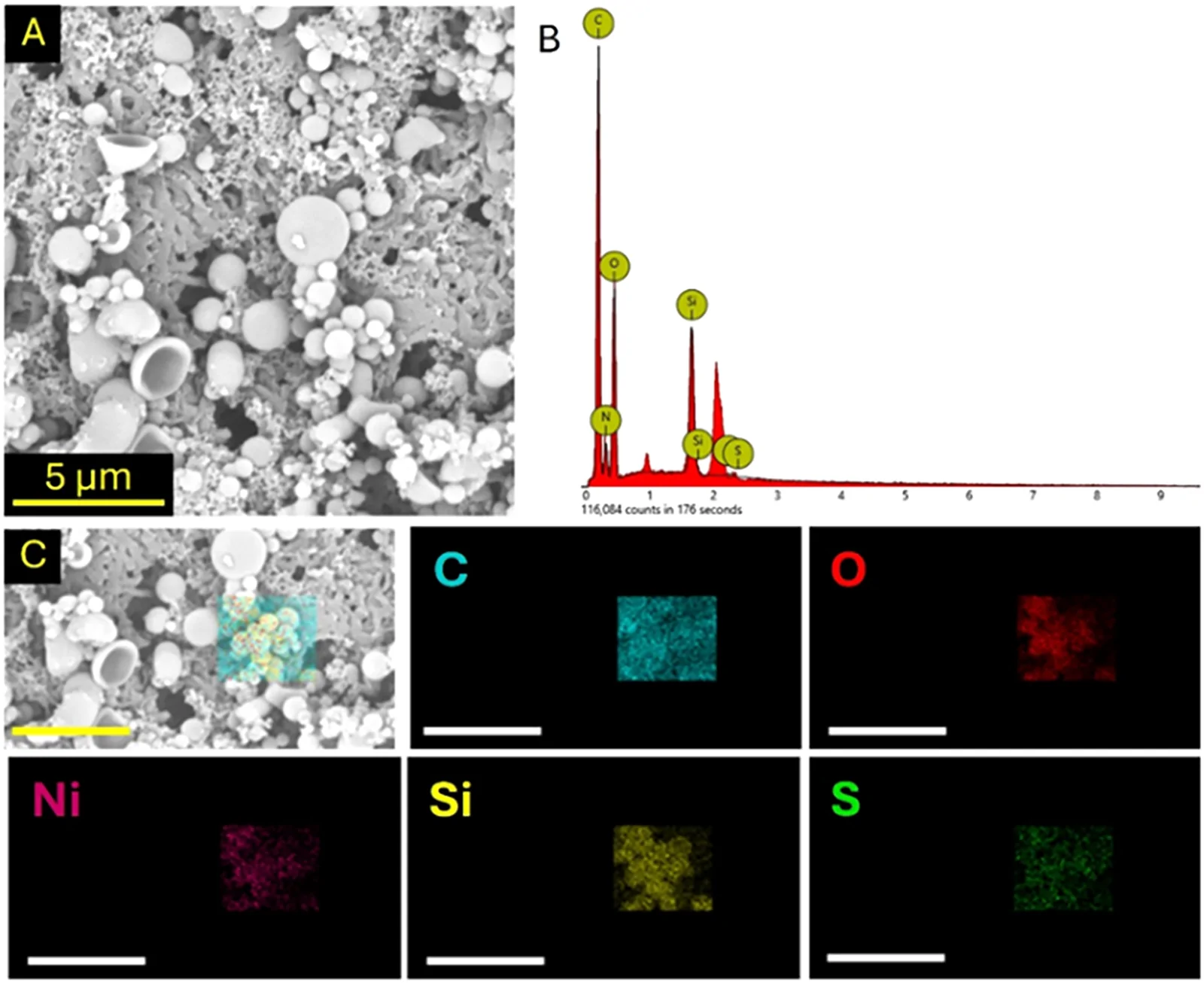
SEM image (A), EDX spectrum (B), and elemental mapping (C) of Laccase-Ny system.

As shown in Table 2, the sulfur content attributable to cysteine and methionine residues in LTV corresponded to 0.25% of the analyzed area. In addition, the EDX spectrum highlighted the predominant presence of carbon, oxygen, nitrogen, and silicon, consistent with the chemical composition of the modified nylon membrane.

**Table 2:** EDX Elemental Mapping Data of Laccase-Ny System

chemical symbol	weight concentration [%]
C	48.93
O	26.88
N	14.62
Si	9.33
S	0.25

### Effect of pH and Temperature on Enzyme Stability

Enzyme stability was evaluated across various pH and temperature conditions to assess the suitability of the platform for biocatalysis. The catalytic performance of free and immobilized LTV was examined at pH 2–9 (Figure 7A). The free enzyme exhibited the highest activity at pH 3, while immobilized LTV displayed a shift in maximal activity toward pH 4.5, suggesting that the microenvironment created by the support affected enzymatic activity.^46^ Moreover, immobilized LTV exhibited higher relative activity under acidic and neutral pH conditions than the free enzyme, demonstrating a notable improvement in enzyme stability upon immobilization. Specifically, immobilized LTV maintained over 80% of its initial activity at pH 3–6, indicating greater tolerance to pH fluctuations. This improved performance may be attributed to the reduced conformational flexibility and enhanced rigidity of the immobilized enzyme, as previously reported.^46^ Figure 7B presents the comparative activity profiles of free and immobilized LTV over the temperature range of 20 to 70 °C. The free enzyme displayed higher catalytic activity at 25 °C, while immobilized LTV exhibited a shift in maximal activity toward 30–40 °C, maintaining 80–90% of its initial activity at up to 45 °C. At higher temperatures, immobilized LTV displayed greater activity than free enzyme. Specifically, at 70 °C, immobilized LTV retained approximately 41.5% of its initial activity, whereas the free enzyme retained only approximately 14.8%. According to a previous report, anchoring to a support may restrict thermal unfolding and shield the enzyme from denaturation, thereby improving the thermal tolerance of the Laccase–Ny system.^60^ The stability of immobilized LTV at different pH values and temperatures was found to be comparable to the values reported by Jasni et al.^26^ and Khanam et al.^30^ in their respective immobilization studies.

**Figure 7:**
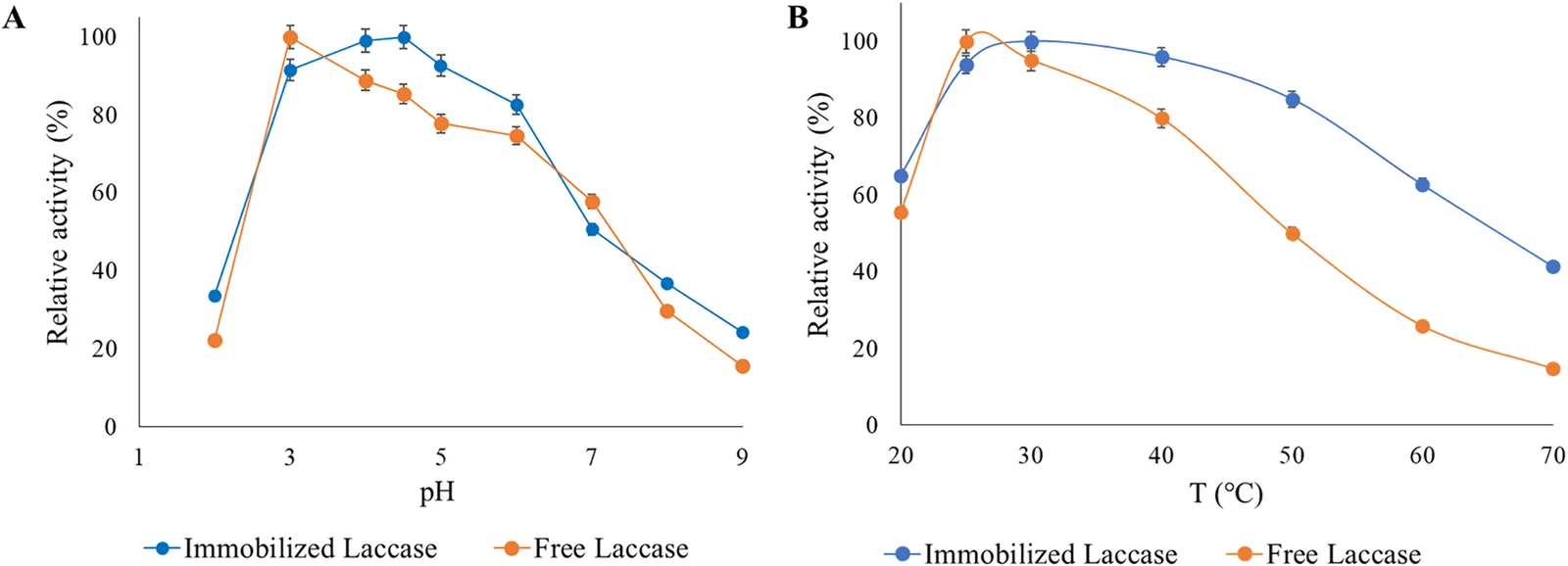
Effects of pH (A) and temperature (B) on the enzymatic activity of free and immobilized laccase. All data are reported as mean ± SD (*n* = 3).

### Biocatalyst Stability and Reusability

The storage stability of free and immobilized LTV was evaluated at 4 °C over a 30-day period (Figure 8A). After 30 days, immobilized LTV retained 82.5% of its initial activity, whereas the free enzyme slowly deteriorated, maintaining only 40% of its original activity. These results are consistent with those of previous immobilization studies^61,62^ and are higher than those reported by Khanam et al.^30^ The enhanced storage stability of immobilized LTV may be ascribed to the interactions between the support and the enzyme, which provide structural reinforcement and reduce its susceptibility to thermal denaturation.^30^ Subsequently, reusability was evaluated as a key parameter for practical application in industrial biocatalysis (Figure 8B). After 10 consecutive operational cycles, immobilized LTV retained approximately 70% of its initial activity, whereas the free enzyme exhibited a significant decrease in activity. This sustained performance is attributed to immobilization procedure employing the binding sites provided by the pyrogallol groups of TA which stabilize LTV and promote the formation of a protective hydration layer around the enzyme–support complex, as previously reported.^30,63,64^

**Figure 8:**
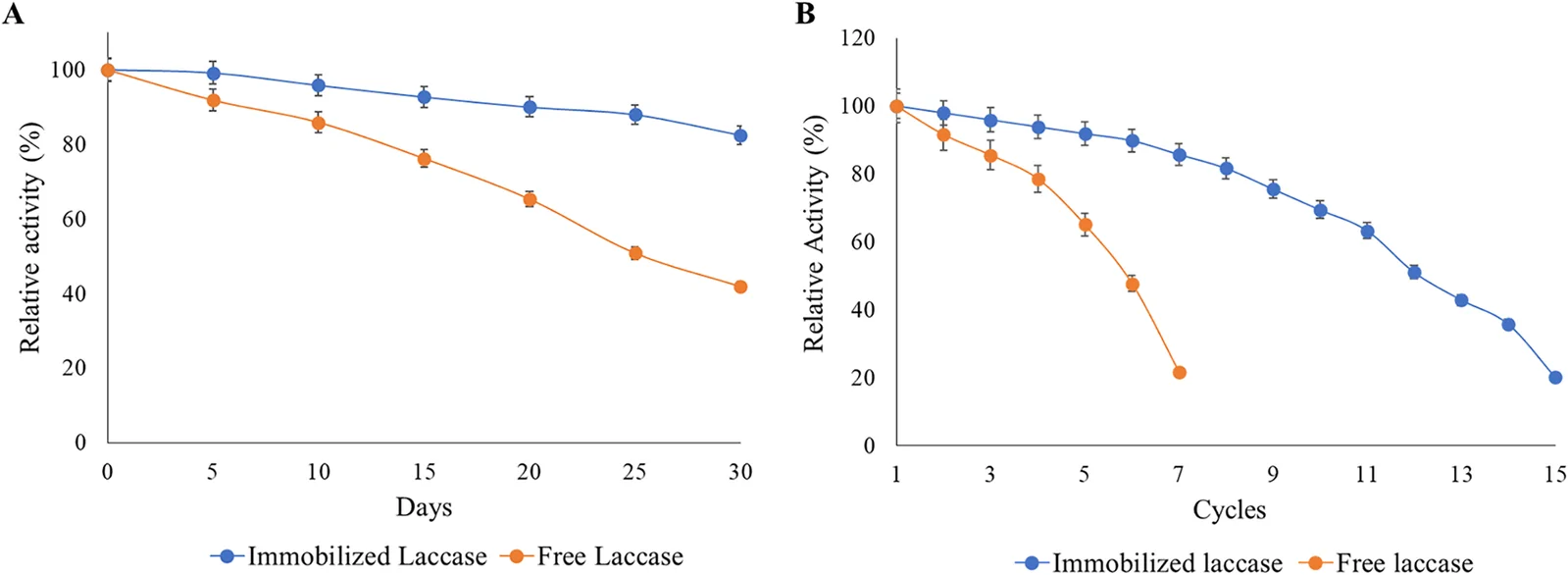
Effect of storage period on stability of free and immobilized laccase under pH 4.5 at 4 °C for 30 days (A) and reusability of free and immobilized laccase in the batch oxidation of ABTS at 25 °C (B). All data are reported as mean ± SD (*n* = 3).

### Kinetic Analysis of Free and Immobilized LTV

Kinetic analysis was conducted to compare the catalytic efficiency of free and immobilized LTV using ABTS as the substrate. The Michaelis–Menten parameters *V*_max_ and *K*_*m*_ were calculated from the intercepts on the reciprocal plot (Figure 9).^48^ *V*_max_ indicates the maximum reaction rate of the enzyme when the substrate fully binds to the active sites, while *K*_*m*_ represents the substrate concentration at which the reaction velocity reaches half of *V*_max_.

**Figure 9:**
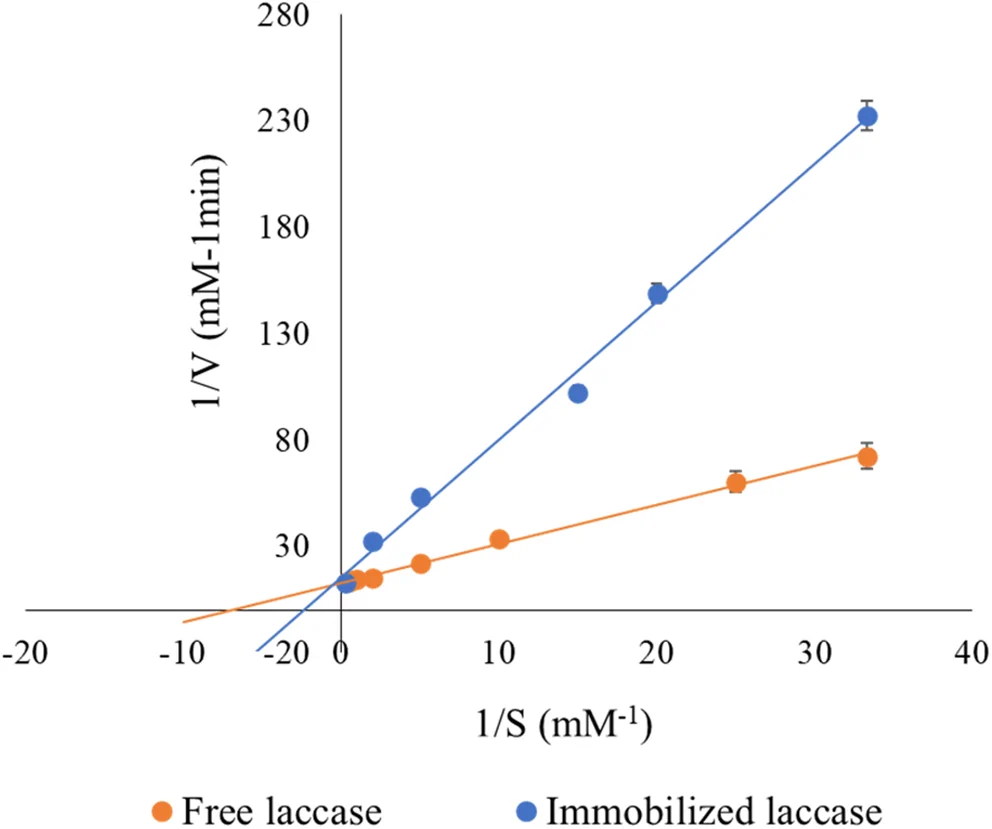
Lineweaver–Burk plots of free and immobilized laccase. All data are reported as mean ± SD (*n* = 3).

A lower ABTS concentration range was used for the free enzyme than for immobilized LTV, aligning with established protocols.^15^ As shown in Table 3, the *V*_max_ of immobilized LTV (0.065 mM/min) was slightly lower than that of the free enzyme (0.077 mM/min), which is attributed to steric hindrance and diffusion limitations imposed by the immobilization matrix. Nevertheless, immobilized LTV maintained a catalytic efficiency comparable to that of the free enzyme.^46^ Conversely, the *K*_*m*_ values were 0.423 and 0.142 mM for immobilized and free LTV, respectively (Table 3), indicating that immobilization on the coated nylon membrane reduced the affinity of LTV toward the substrate. This reduction in affinity is likely due to localized alterations in the microenvironment, diffusion barriers, and reduced enzyme flexibility caused by immobilization. Additionally, the binding of LTV to the coated nylon membrane may reduce the number of accessible active sites.^15,46,65^ Nevertheless, immobilized LTV exhibited high catalytic activity despite a reduced *V*_max_ and increased *K*_*m*_ compared to its free counterpart.

**Table 3:** Kinetic Parameters of Free and Immobilized Laccase (Laccase-Ny System)^a^

	*K_m_* (mM)	*V*_max_ (mM/min)
free laccase	0.142 ± 0.002	0.077 ± 0.005
Laccase-Ny system	0.423 ± 0.004	0.065 ± 0.002

a All data are reported as mean ± SD (*n* = 3).

### Conformational Analysis of Free and Immobilized LTV

The effect of immobilization on the conformational structure of LTV was investigated via Raman spectroscopy, as shown in Figure 10. The Raman spectrum of the pristine nylon membrane (black curve) exhibited the well-known characteristic vibrational peaks, such as the amide I band (∼1626 cm^–1^, C=O stretching) and the CH_2_ scissoring mode (approximately1440–1465 cm^–1^). Additional backbone-related vibrations appeared in the amide III region (1290 cm^–1^) and in the C–C/C–N stretching region below 1200 cm^–1^.^66^ These features confirmed the expected spectral signature of nylon and served as a reference background for subsequent measurements. By contrast, the Raman spectrum of the coated nylon membrane (red curve) exhibited a strong peak in the 1600 cm^–1^ region, corresponding to the aromatic ring C=C stretching modes of TA.^67^ No other distinct signals from TA or APTES were detected. Conversely, the Raman spectrum of the laccase–Ny system (blue curve) provided spectroscopic evidence that the protein retained a well-defined secondary structure upon immobilization onto the modified nylon surface. Specifically, the amide I region (1600–1700 cm^–1^), dominated by the C=O stretching vibration of the peptide bond, exhibited a pronounced contribution in the 1610–1630 cm^–1^ range, characteristic of β-sheet-rich structures, and minor components extending toward the 1670–1690 cm^–1^ region, attributable to β-turns. Only a weak contribution was observed in the 1650–1658 cm^–1^ region, indicating limited α-helical content. The absence of a dominant band at ∼1645 cm^–1^, typically associated with random coil conformations, suggests that the immobilization process did not induce substantial protein unfolding.^68,69^ Additional confirmation was provided by the amide III region (1220–1300 cm^–1^), where bands in the 1230–1250 cm^–1^ range supported the predominance of β-sheets, while contributions within the 1270–1290 cm^–1^ range were associated with β-turns and minor α-helical segments. The agreement between the amide I and III regions indicated that the LTV backbone retained an ordered, native-like conformation upon immobilization.^70,71^ Collectively, these results provide strong evidence that LTV immobilized onto a modified nylon membrane maintains its native, β-sheet–dominated secondary structure, confirming the modified nylon membrane as a stable substrate for enzyme immobilization.

**Figure 10:**
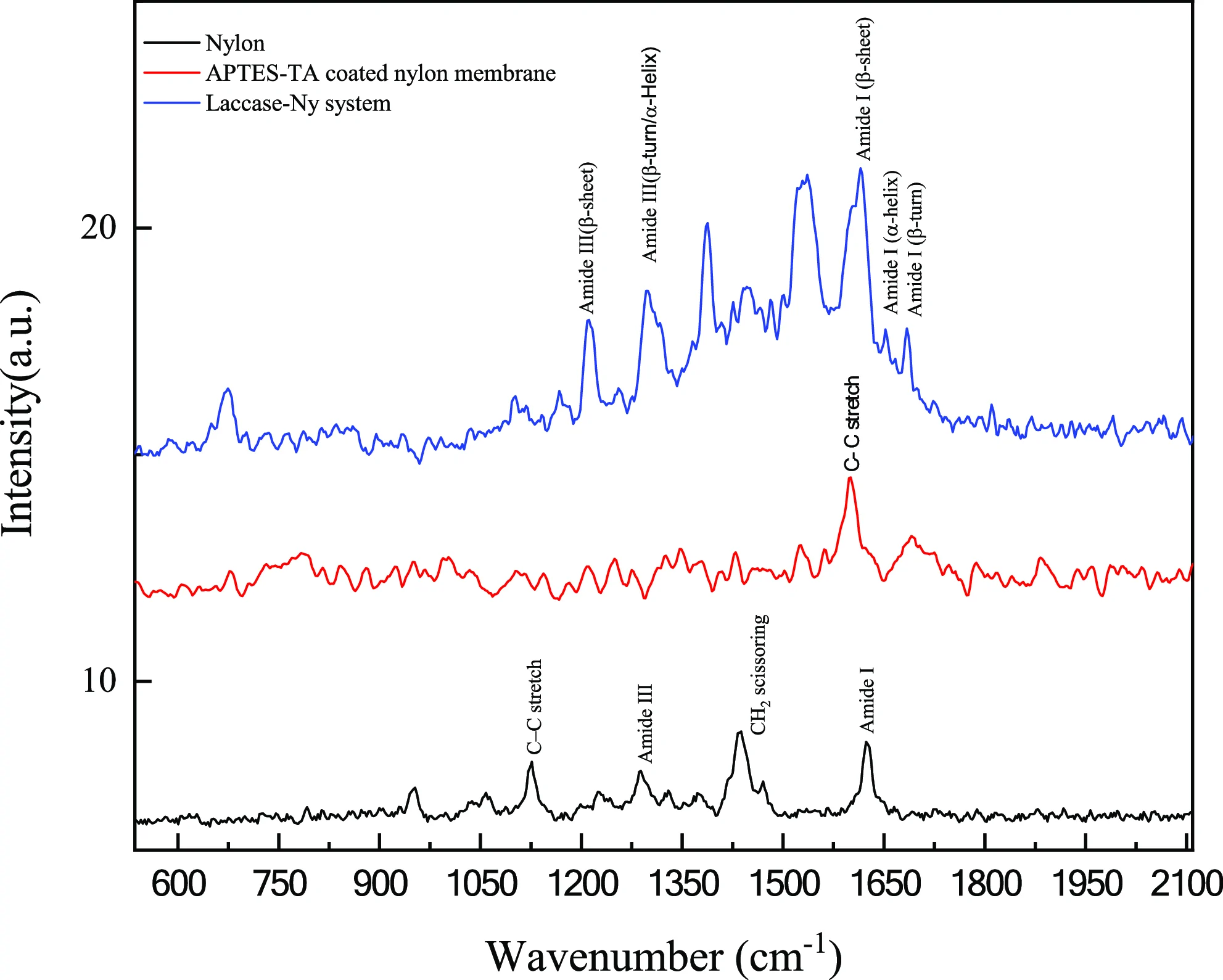
Raman spectra of nylon (black), APTES-TA coated nylon membrane­(red), and Laccase-Ny system (blue).

### Fluorescence Analysis

Fluorescence analysis provided additional information about LTV structural changes and valuable insights into the microenvironment of chromophores. LTV exhibits intrinsic fluorescence due to aromatic amino acids such as tryptophan, phenylalanine, and tyrosine.^72,73^ As shown in Figure 11A, the fluorescence intensity of LTV decreased upon immobilization, suggesting that immobilization altered the surroundings of aromatic amino acids such as tryptophan. Thermal fluorescence analysis was performed at 310 nm (λ_max_) over the temperature range of 25–45 °C (Figure S4). An increase in temperature caused a progressive reduction in fluorescence intensity for free and immobilized LTV. However, immobilized LTV exhibited a lower intensity than the free enzyme, indicating enhanced structural stability under thermal stress. Subsequently, extrinsic fluorescence analysis was performed using the ANS probe, which targets hydrophobic pockets within proteins. Conformational changes in these regions typically cause variations in emission intensity and a blue shift in the emission wavelength. Structural studies comparing free and immobilized LTV via ANS fluorescence analysis (Figure 11B) revealed a market increase in fluorescence intensity following immobilization. This finding suggests that immobilization induced structural changes in LTV, increasing the exposure of surface hydrophobic pockets.

**Figure 11:**
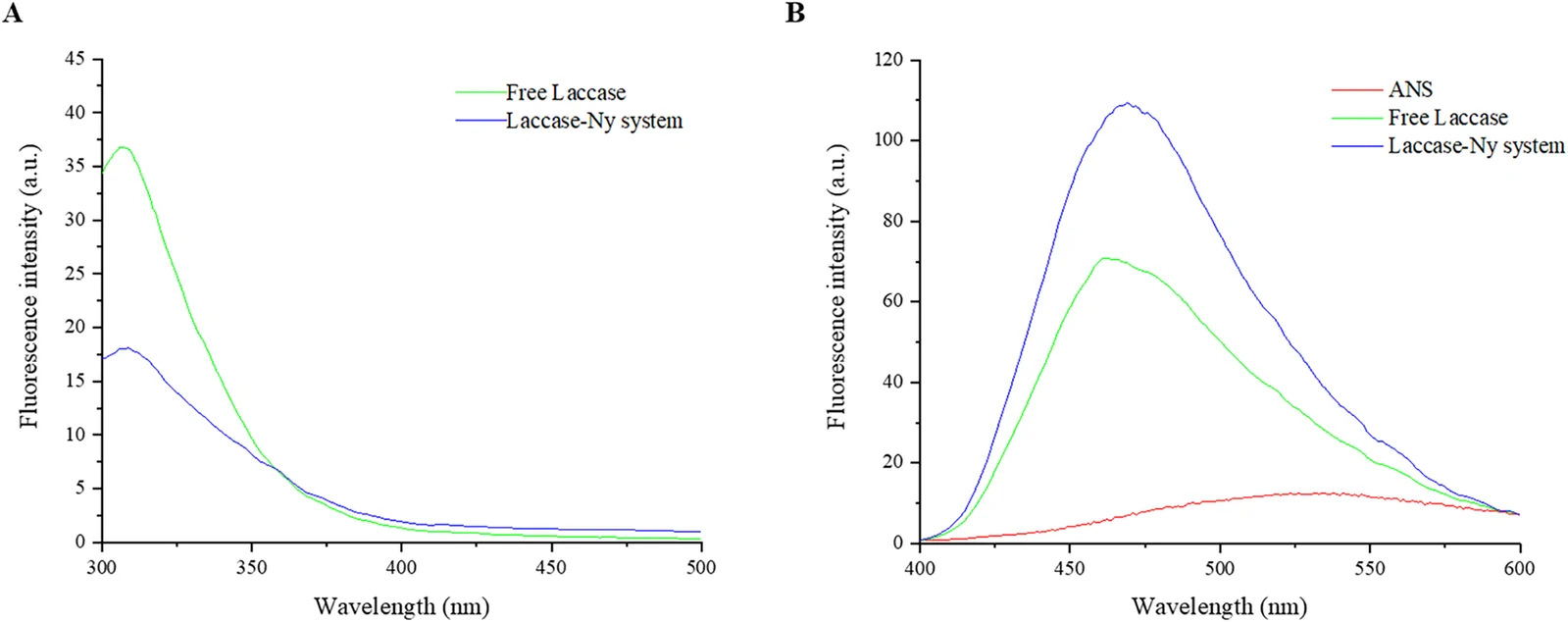
Emission spectrum of the intrinsic (A) and extrinsic (B) fluorescence emission spectrum of free and immobilized enzyme. The spectra were recorded in acetate buffer (0.1 M, pH 4.5) at 25 °C, containing an enzyme concentration of 0.3 mg/mL.

### Dye Decolorization Application

The decolorization of MB and MR dyes (Figure S5) was investigated over time using free and immobilized LTV. Decolorization was assessed via HPLC-UV detection by monitoring changes in peak area at the characteristic absorption wavelengths of each dye. As shown in Figure 12A, decolorization proceeded rapidly and was essentially complete within 24 h. The free enzyme achieved dye removal efficiencies of 81.8 and 78.5% for MB and MR, respectively, with immobilized LTV achieving corresponding values of 96.0 and 97.0% (Figure 12B). By comparison, the pristine nylon membrane removed 58.3% of MB and 51.8% of MR after 24 h. These findings suggest a dual decolorization mechanism involving physical absorption onto the nylon membrane and other transformations aided by immobilized LTV. Similar phenomena were reported by Nouaa et al.^46^ and Hürmüzlü et al.,^74^ who observed that dye removal using LTV immobilized on LDH/alginate composite beads and chitosan/halloysite supports, respectively, involved coupled adsorption and biodegradation processes. Overall, these results demonstrate that immobilized LTV, with its enhanced activity, stability, and catalytic efficiency compared to the free enzyme, represents a promising platform for industrial dye decolorization.

**Figure 12:**
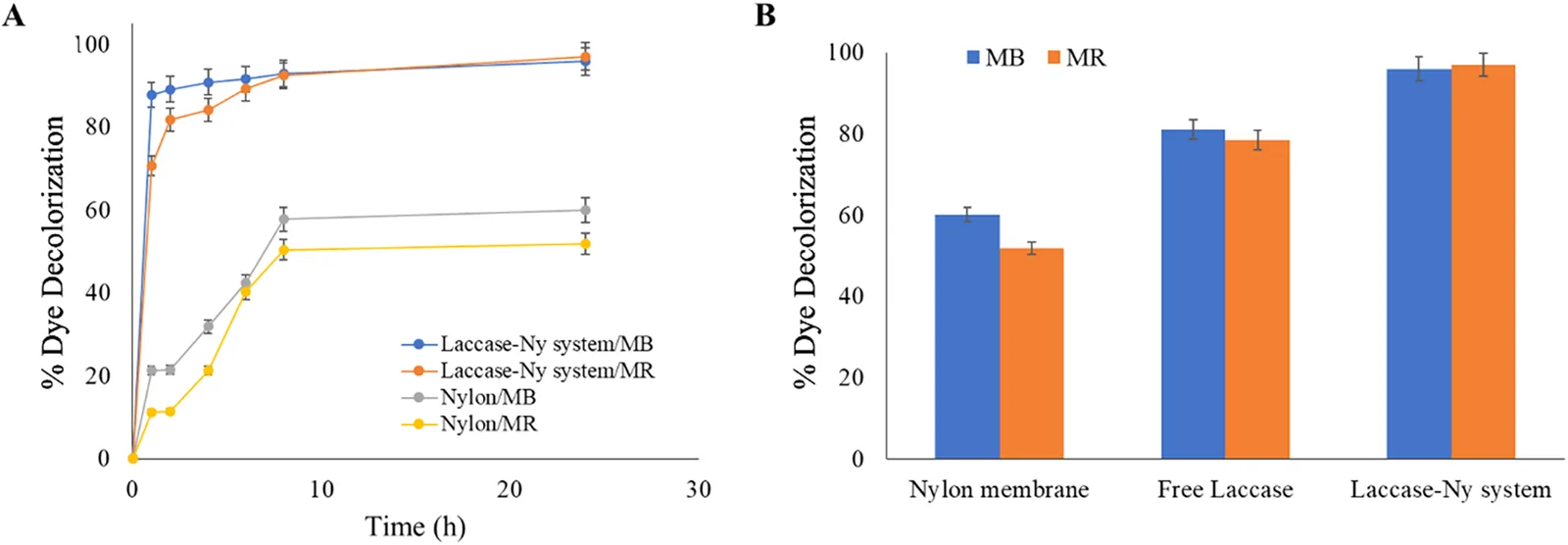
Kinetic effect on the removal of MB and MR dye with nylon membrane and Laccase-Ny system (A) and decolorization rate of MB and MR by nylon, free laccase, and Laccase-Ny system (B). All data are reported as mean ± SD (*n* = 3).

## Conclusion

LTV was successfully immobilized onto a nylon membrane modified with a codeposited APTES–TA coating. A key novelty of this work lies in transforming the −OH groups of TA into −COOH groups within the coating matrix followed by a laccase immobilization procedure. This approach differs from earlier studies, in which enzymes were anchored mainly through Schiff base or Michael addition reactions. The RSM–DoE approach (Box–Behnken design) was employed to optimize immobilization conditions (e.g., pH, enzyme concentration, TA:APTES ratio, and reaction time), achieving an optimal immobilization yield of 67.3%. Compared to the free enzyme, immobilized LTV exhibited significantly enhanced thermal and pH stability, retaining over 70% activity after 10 cycles. Furthermore, immobilized LTV maintained a catalytic efficiency comparable to that of its free counterpart while retaining its native, β-sheet-dominated secondary structure. ANS fluorescence analysis revealed that immobilization induced a structural change in the enzyme, increasing the exposure of surface hydrophobic pockets. The resulting Laccase–Ny system was validated through the successful decolorization of MB and MR dyes, confirming its bioremediation potential. This novel Laccase–Ny system provides a valuable, efficient biocatalyst platform for potential wastewater treatment, synthetic processes, and other industrial applications. Moreover, the inherent antimicrobial activity of the TA coating^75,76^ could mitigate biofouling, thereby extending the operational lifetime of the membrane.

## Supplementary Material



## Notes

Additional experimental design tables (Tables S1–S5); ATR-FTIR spectrum of nylon and APTES-TA coated nylon membrane (Figure S1); ATR-FTIR spectrum of nylon membrane and Laccase-pristine nylon membrane (Figure S2); Raman spectra of free and immobilized laccase (Figure S3), thermal fluorescence data (Figure S4); molecular structures of dyes (Figure S5); oxidation of ABTS in the presence of laccase (Figure S6); HPLC-UV chromatograms of dyes monitored at 665 and 530 nm before and after the reaction mediated by Laccase-Ny system (Figure S7); HPLC-UV chromatograms of dyes monitored over time at 665 nm (Figure S8); HPLC-UV chromatograms of dyes monitored over time at 530 nm (Figure S9); pathway of MB transformation aided by laccase (Figure S10); pathway of MR transformation aided by laccase (Figure S11); ESI-MS spectrum of MB and resulting products after Laccase-Ny system treatment (Figure S12); ESI-MS spectrum of MR resulting products after Laccase-Ny system treatment (Figure S13); oxidation reaction of catechol in benzoquinone by Laccase-Ny system (Figure S14); HPLC-UV chromatograms of catechol: before and after the oxidation reaction mediated by Laccase-Ny system (Figure S15) (PDF)
