# Supplementary material for: Laccase Immobilization on Tannic Acid–Coated Nylon Membrane for Dye Decolorization
Source: ACS Omega. 2026 Aug 5;11(32):47346–59. doi: 10.1021/acsomega.6c00521 (PMC13491474; doi:10.1021/acsomega.6c00521)
Supplement: Supplementary file 1 [file ao6c00521_si_001.pdf]

Laccase immobilization on tannic acid–coated nylon membrane for dye decolorization

Claudia Sciacca,[a] Nunzio Cardullo,[a] Marcello Condorelli,[a] Libera Vitiello,[b]

*Andrea A. Scamporrino,[b] Sabrina C. Carroccio,[b] Vera Muccilli [a,b]**

[a] University of Catania, Department of Chemical Sciences, V. le A. Doria 6, 95125, Catania (Italy)

[b] Institute for Polymers, Composites and Biomaterials CNR, Via P. Gaifami 18, 95126, Catania (Italy)

Corresponding author: vera.muccilli@unict.it

Table S1. Conditions applied in the single-factor experiments for the optimization of immobilization reaction conditions.

Variables	*Factors*	*Levels*	Y_1
X_1	*Buffer pH immobilization*	*3, 4, 5*, 6.5, 8.5*	*LTV immobilized amount (%w/w)*
X_2	*LTV concentration (mg/mL)*	*0.3, 0.6, 0.9*, 1.2, 1.5*	
X_3	*Ratio TA:APTES*	*1:1, 2:1, 4:1*, 8:1,12:1,*	
X_4	*Reaction time (h)*	*8, 16, 24*, 32, 48*	

*Fixed value. The ($\%^{w}/_{w}$) referred to the weight of TA-APTES coated nylon membrane.

Table S2. Results of the single-factor experiment for immobilizing LTV onto nylon membrane.

pH (X_1)	*LTV amount ($\%^{w}/_{w}$)*	*LTV Conc.(mg/mL) (X_2)*	*LTV amount ($\%^{w}/_{w}$)*	*Ratio TA:APTES (X_3)*	*LTV amount ($\%^{w}/_{w}$)*	*Reaction time (h) (X_4)*	*LTV amount ($\%^{w}/_{w}$)*
3	5.43 ± 0.09^{h}	0.3	9.16 ± 0.23^{e}	1:1	2.88 ± 0.03^{i}	8	2.87 ± 0.04^{i}
4	8.25 ± 0.14^{f}	0.6	20.30 ± 0.31^{a}	2:1	12.40 ± 0.31^{d}	16	6.74 ± 0.07^{g}
5	14.01 ± 0.42^{c}	0.9	14.01 ± 0.42^{c}	4:1	14.01 ± 0.42^{c}	24	14.01 ± 0.42^{c}
6.5	2.61 ± 0.05^{i}	1.5	13.70 ± 0.38^{c}	8:1	18.40 ± 0.44^{b}	32	$0.83 \pm 0.02^{j,k}$
8.5	0.23 ± 0.01^{k}	2	2.65 ± 0.03^{i}	12:1	1.30 ± 0.02^{j}	48	0.55 ± 0.01^{k}

Data are reported as means ± standard deviations. Different letters in the same column indicate significant differences (Tukey's test $p<0.05$). The ($\%^{w}/_{w}$) referred to the weight of TA-APTES-coated nylon.

Table S3. Preliminary ranges and coded levels of the selected independent variables.

Independent variables	*Code*	*Coded levels*		
		-1	*0*	*+1*
pH	X_1	3	4	5
LTV concentration (mg/mL)	X_2	0.3	0.6	0.9
Ratio TA/APTES	X_3	2:1	4:1	8:1
Reaction time (h)	X_4	8	16	24

Table S4. Experimental design and result of the % amount of LTV.

Run	*Independent variables*				*Dependent variables*
	Reaction conditions				*Experimental values*
	X_1	*X_2*	*X_3*	*X_4*	*Y_1*
	pH	*(LTV conc., mg/mL)*	*(Ratio)*	*(Reaction time, h)*	*(LTV amount $\%^{w}/_{w}$)*
1	*4*	*0.9*	*8:1*	*16*	*11.60 ± 0.81*
2	*3*	*0.3*	*4:1*	*16*	*10.20 ± 0.62*
3	*4*	*0.3*	*4:1*	*8*	*3.50 ± 0.07*
4	*5*	*0.6*	*2:1*	*16*	*10.80 ± 0.80*
5	*3*	*0.6*	*2:1*	*16*	*14.10 ± 1.12*
6	*4*	*0.3*	*8:1*	*16*	*4.61 ± 0.36*
7	*3*	*0.9*	*4:1*	*16*	*15.22 ± 1.28*
8	*5*	*0.6*	*4:1*	*8*	*0.91 ± 0.08*
9	*4*	*0.6*	*8:1*	*24*	*9.34 ± 0.75*
10	*3*	*0.3*	*8:1*	*16*	*4.40 ± 0.31*
11	*5*	*0.6*	*8:1*	*16*	*24.80 ± 1.93*
12	*5*	*0.6*	*4:1*	*24*	*13.93 ± 1.14*
13	*4*	*0.6*	*4:1*	*16*	*6.20 ± 0.07*
14	*3*	*0.6*	*4:1*	*24*	*10.01 ± 0.98*
15	*4*	*0.6*	*8:1*	*8*	*3.93 ± 0.35*
16	*5*	*0.9*	*4:1*	*16*	*11.15 ± 0.81*
17	*4*	*0.3*	*4:1*	*24*	*15.17 ± 1.29*
18	*4*	*0.9*	*2:1*	*16*	*15.40 ± 1.30*
19	*4*	*0.9*	*4:1*	*8*	*0.32 ± 0.02*
20	*3*	*0.6*	*4:1*	*8*	*0.32 ± 0.03*
21	*4*	*0.6*	*2:1*	*24*	*17.53 ± 1.54*
22	*4*	*0.6*	*4:1*	*16*	*6.58 ± 0.08*
23	*5*	*0.3*	*4:1*	*16*	*21.37 ± 1.45*
24	*5*	*0.6*	*2:1*	*16*	*9.81± 1.11*

25	*5*	*0.6*	*8:1*	*16*	*21.23 ± 1.44*
26	*4*	*0.6*	*4:1*	*16*	*6.58 ±0.09*
27	*3*	*0.9*	*4:1*	*16*	*15.80± 1.27*

The (%$^{w}/_{w}$) referred to the weight of APTES-TA coated nylon membrane.

Table S5. Comparison between actual-by-predicted values of immobilization laccase obtained at optimal conditions.

Responses	*LTV immobilized amount (%$^{w}/_{w}$)*
Measured	*31.9 ± 2.1*[a]
Predicted	*36.1*[a] *(29.6 – 42.6)*

Data are reported as means ± standard deviations, and letters in the same column indicate significant differences (Tukey's test p <0.05).

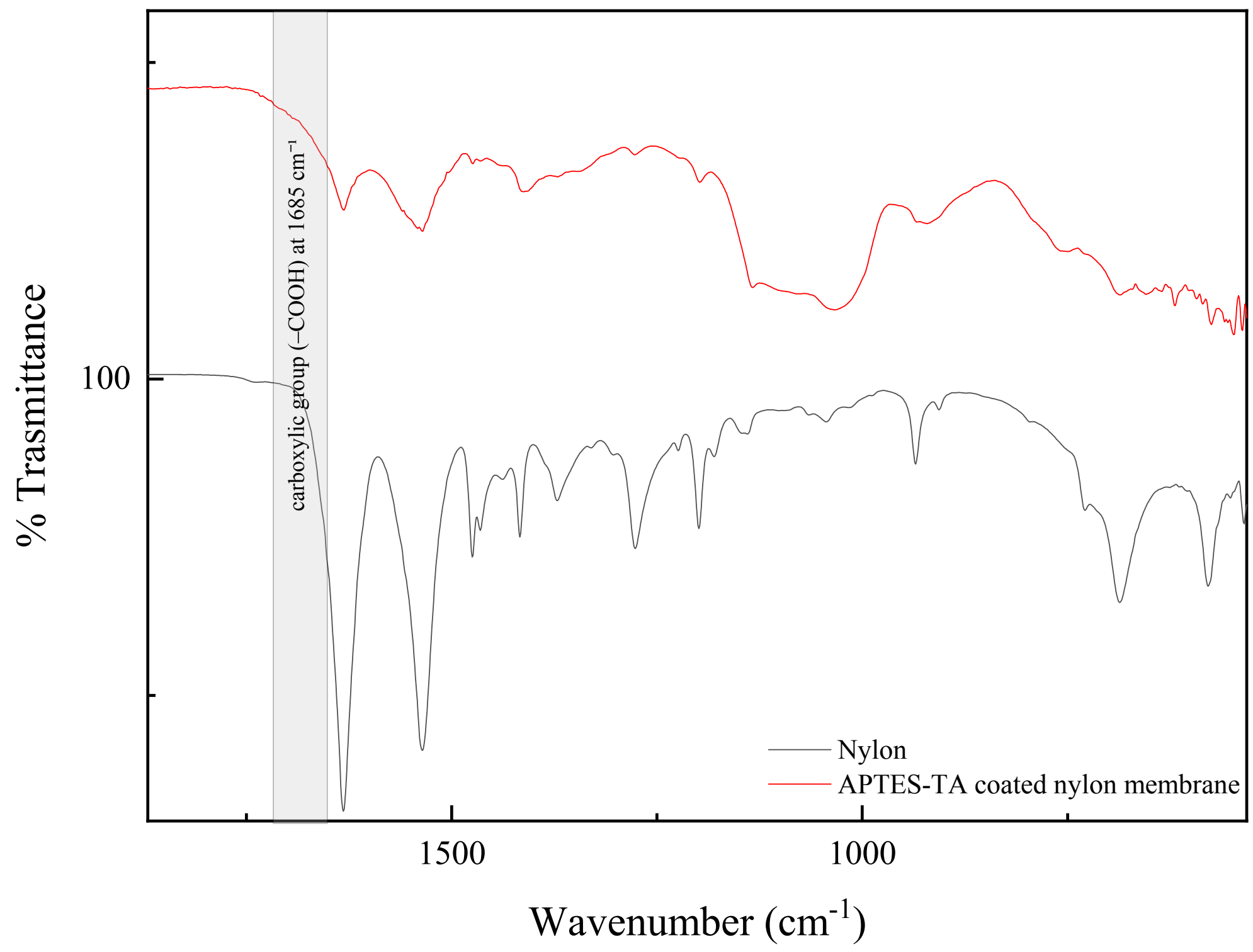

Figure S1. ATR-FTIR spectrum of nylon membrane (black) and APTES-TA coated nylon membrane without baseline correction (red).

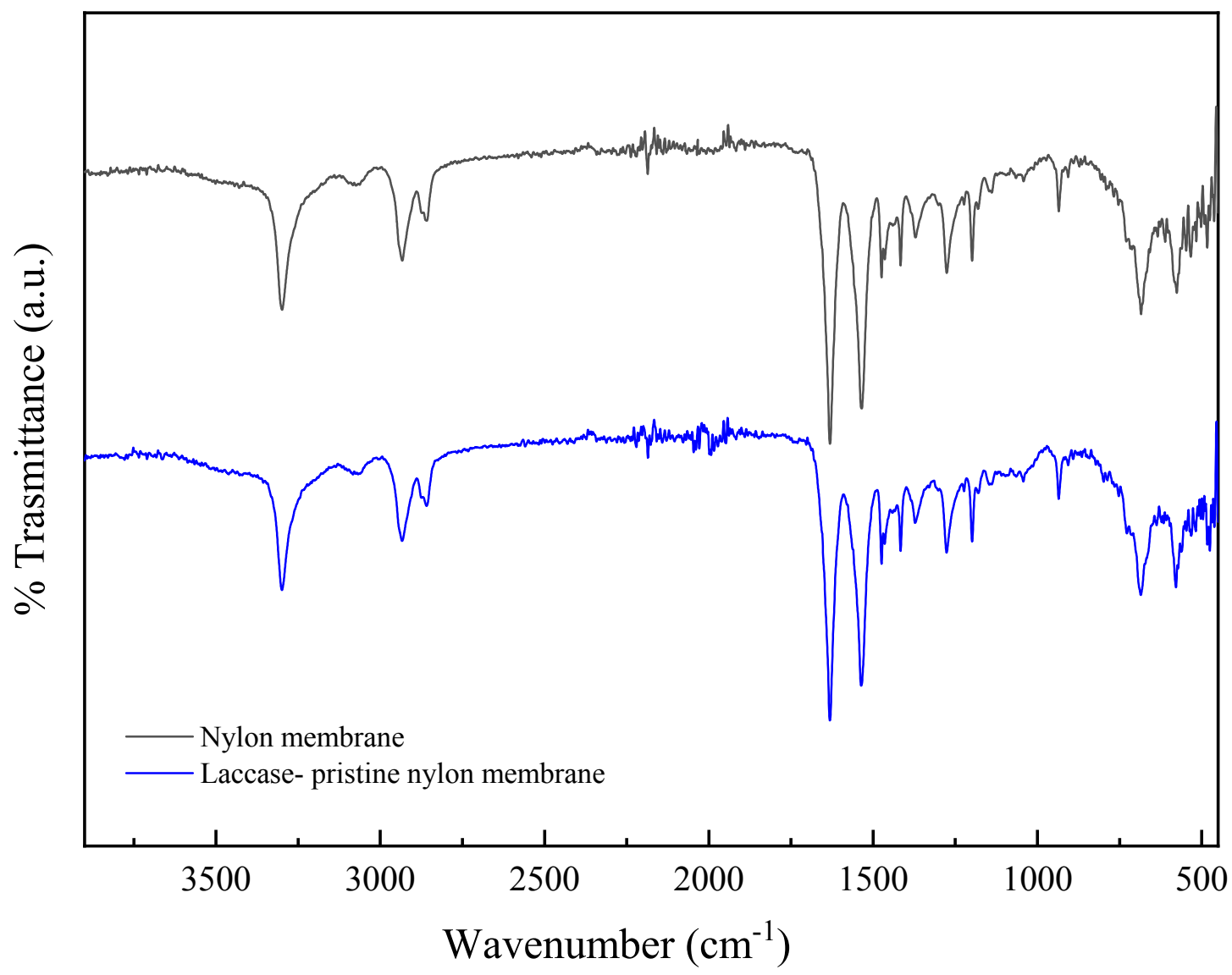

Figure S2. ATR-FTIR spectrum of nylon membrane (black), Laccase - pristine nylon membrane (blue).

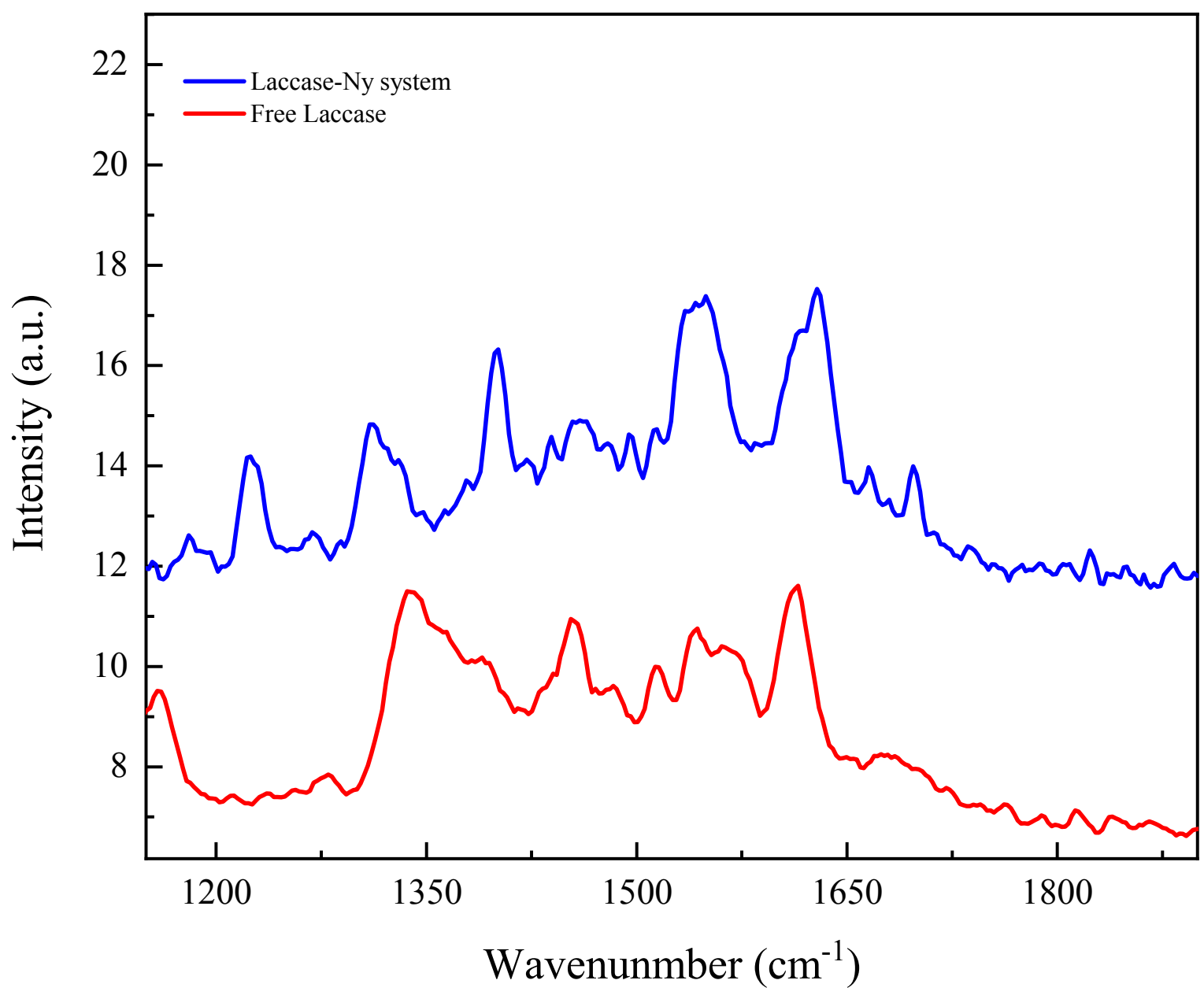

Figure S3. Raman spectra of Laccase powder (red) and Laccase-Ny system (blu).

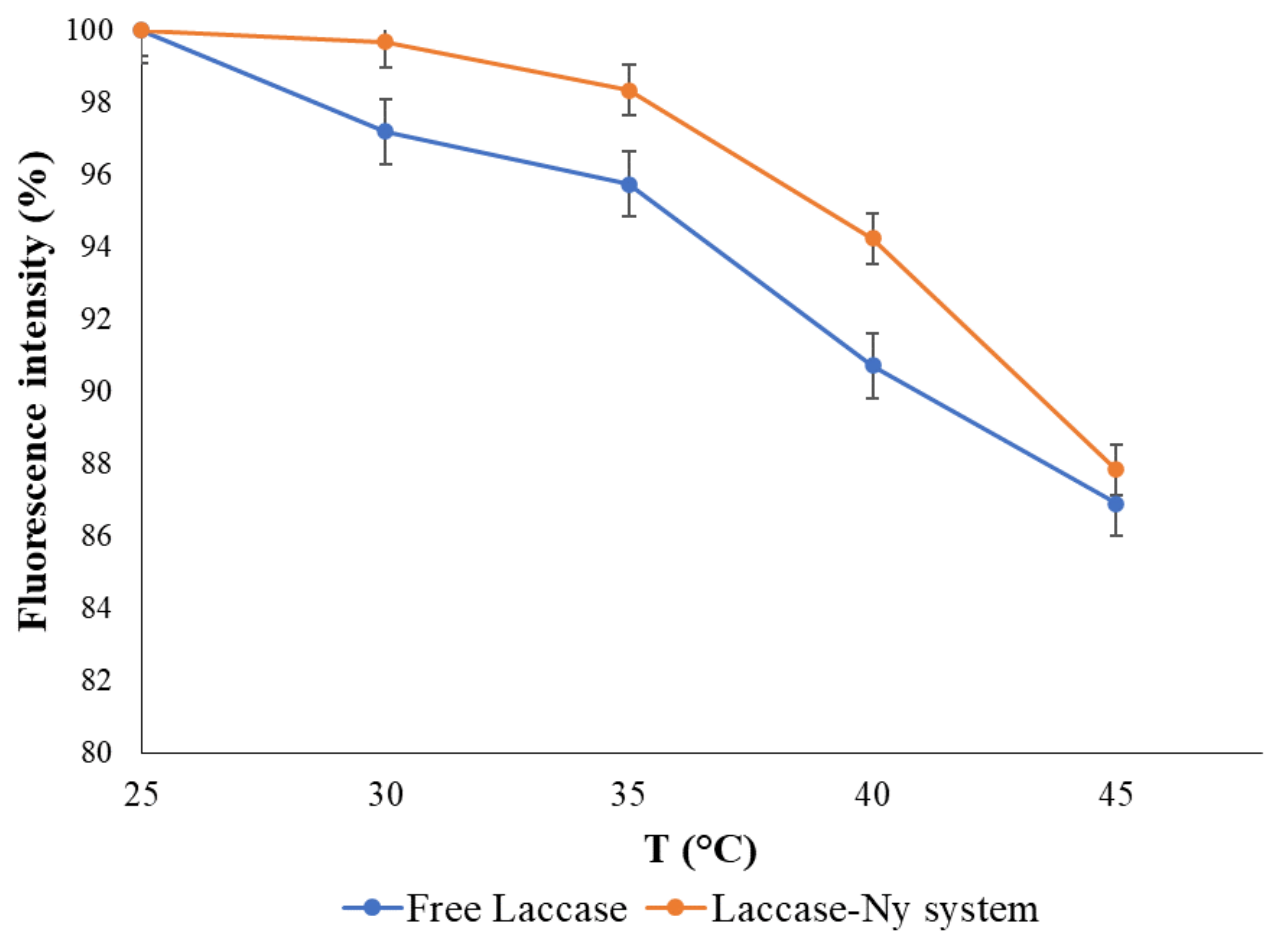

Figure S4. Thermal fluorescence analysis of free and immobilized LTV at 310 nm (λ_{max}) over the temperature ranges of 25 - 45 °C.

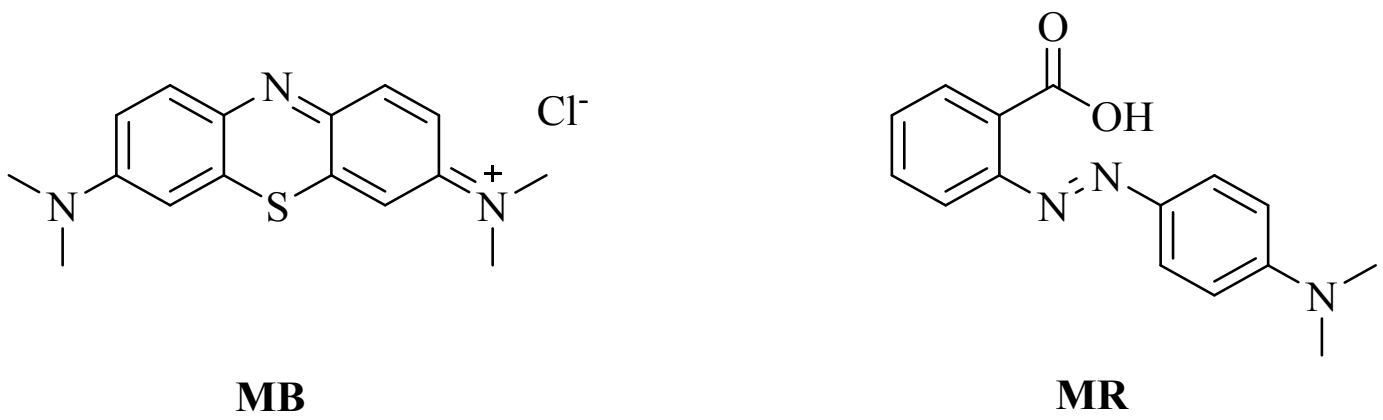

Figure S5. Molecular structure of methylene blue (MB) and methyl red (MR).

Reaction mechanism of laccase on ABTS, Methylene blue (MB) and Methyl red (MR)

Figure S6 shows the laccase-mediated oxidation of ABTS. As reported in literature, ABTS undergoes an initial one-electron oxidation to form the relatively stable radical cation $ABTS^{\cdot+}$ (blue coloured radical). In a subsequently step, the radical is further oxidized to the dication $ABTS^{2+}$, red-coloured species less stable.[1,2]

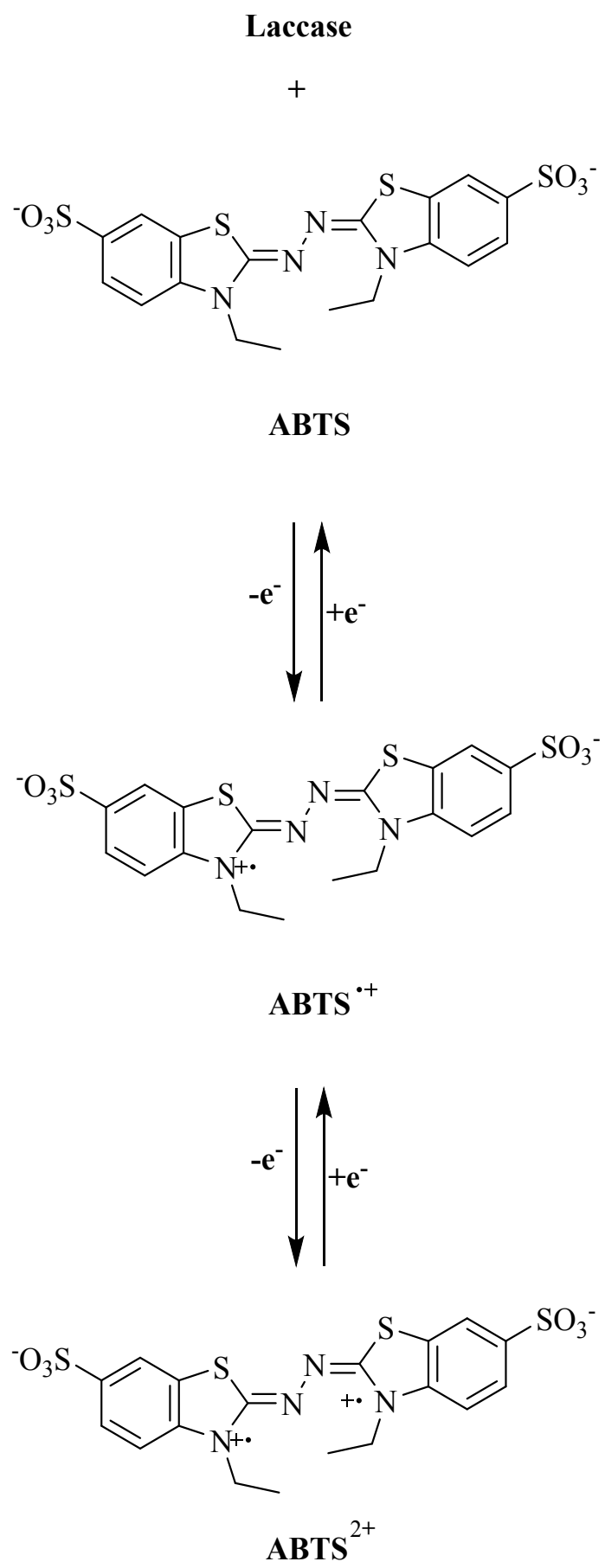

Figure S6. Oxidation of ABTS in the presence of laccase. [1,2]

Figure S7 reports the HPLC-UV chromatograms of a mixture of MB and MR before and after 24 h of treatment with Laccase-Ny system. A progressive decrease in the peak areas of both dyes and the appearance of a new signal at approximately 10 min were observed. Detection was performed at the maximum absorption wavelengths of the two dyes, specifically 665 nm for MB and 530 nm for MR.

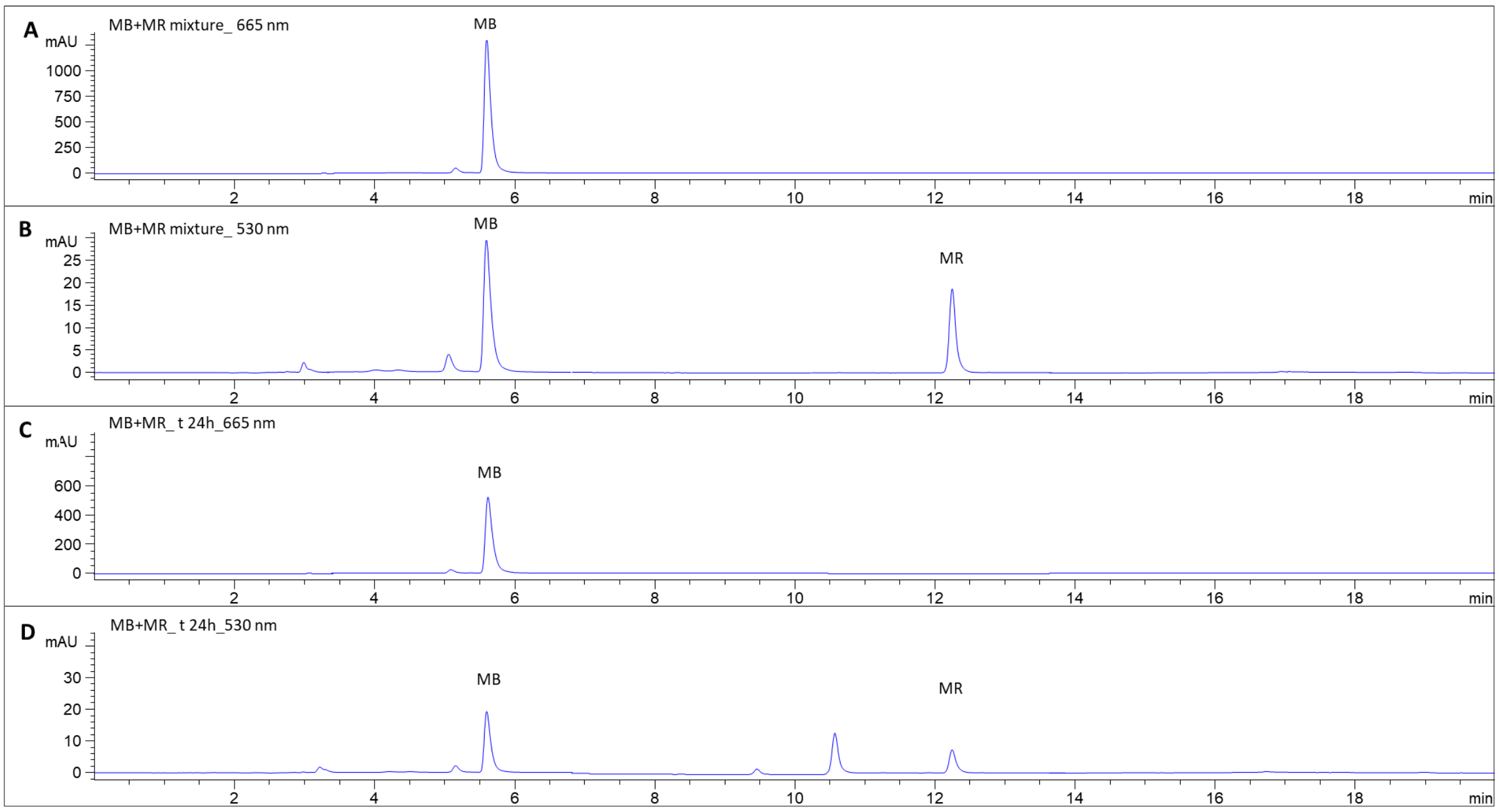

Figure S7. HPLC-UV chromatograms of dyes monitored at 665 and 530 nm: (A and B) before and (C and D) after the reaction mediated by the Laccase-Ny system.

As a control experiment to assess possible dye decolorization over 24 h, a mixture of both dyes, in the absence of the immobilized LTV, was monitored under identical conditions. As shown in **Figures S8 and S9**, no additional peaks were detected, and the only chromatographic peaks corresponded to the original dyes' signals.

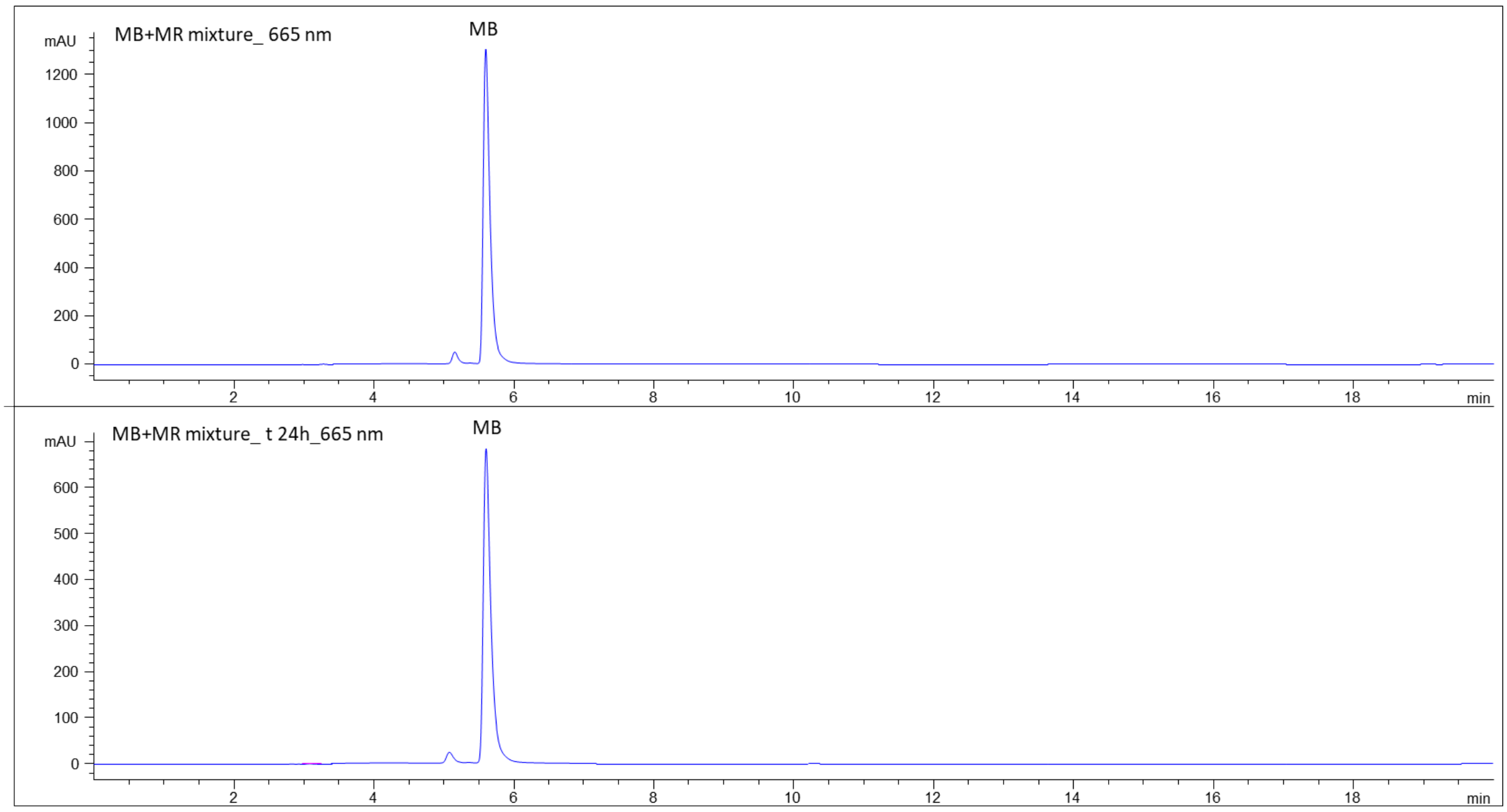

Figure S8. HPLC-UV chromatograms of dyes monitored over time at 665 nm.

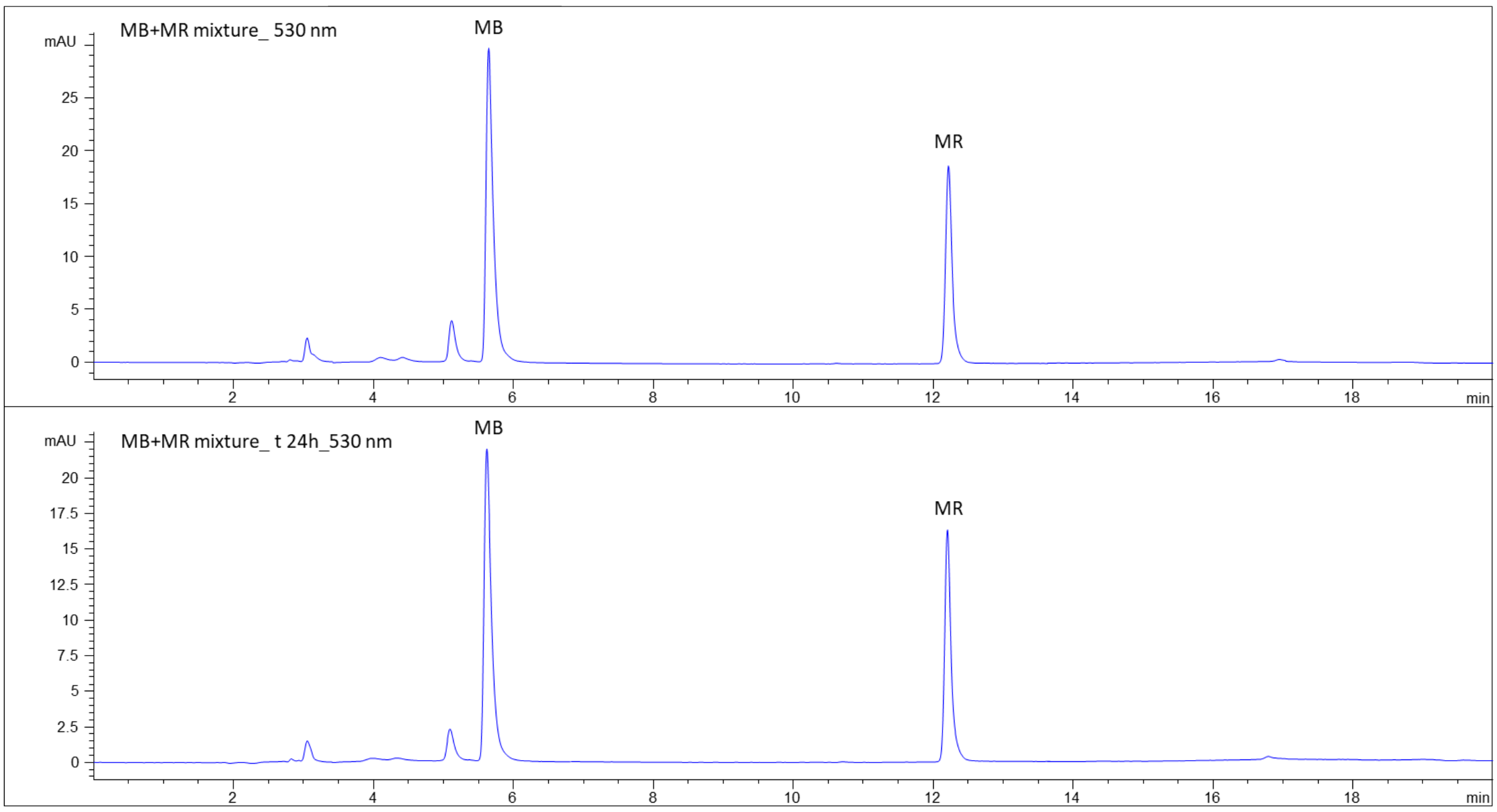

Figure S9. HPLC-UV chromatograms of dyes monitored over time at 530 nm.

Figure S10 reports the proposed laccase-mediated pathway for methylene blue decolorization. As reported by *Sanchez-Alvarez et al.*,[3] the mechanism is assumed to begin with successive oxidative demethylation steps that convert MB into Azure A, Azure B, and Azure C, followed by further demethylation to yield thionine. *Kangli et al.*[4] reported that reactive oxygen species generated by laccase can disrupt the molecular structure of methylene blue, converting it into

lower-molecular-weight compounds. In particular, the C–N and C–S bonds in the dye molecule are susceptible to oxidative cleavage, yielding smaller products that can be further oxidized.

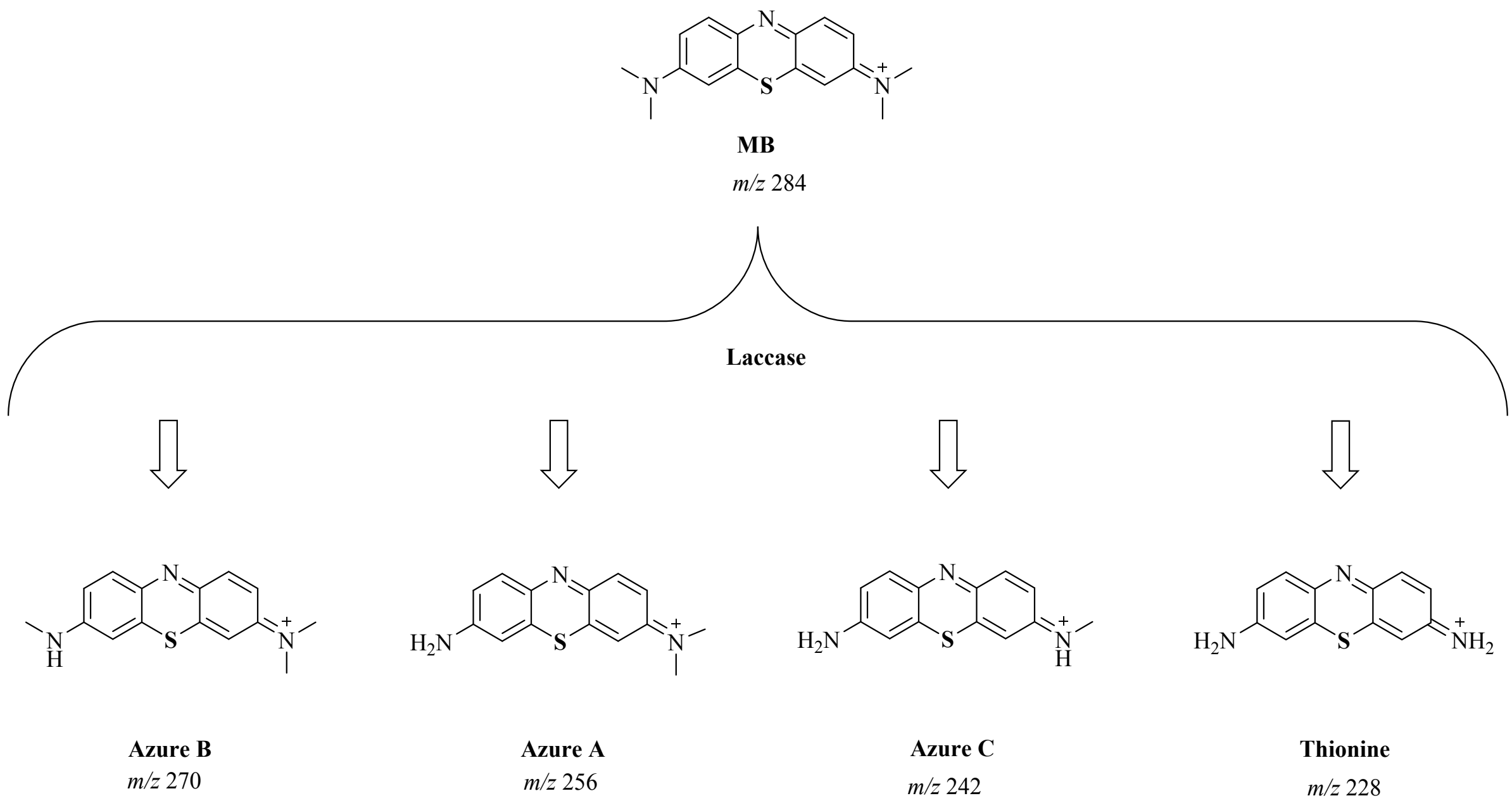

Figure S10. Pathway of methylene blue (MB) transformation aided by laccase.[3,4]

The laccase-mediated pathway for methyl red (MR) decolorization, illustrated in **Figure S11**, indicates that the process begins with the asymmetric cleavage of the azo bond, leading to the formation of two main intermediate products: 2-amino benzoic acid and *N,N*-dimethyl-*p*-phenylendiamine, which may undergo further transformation reactions.[5,6]

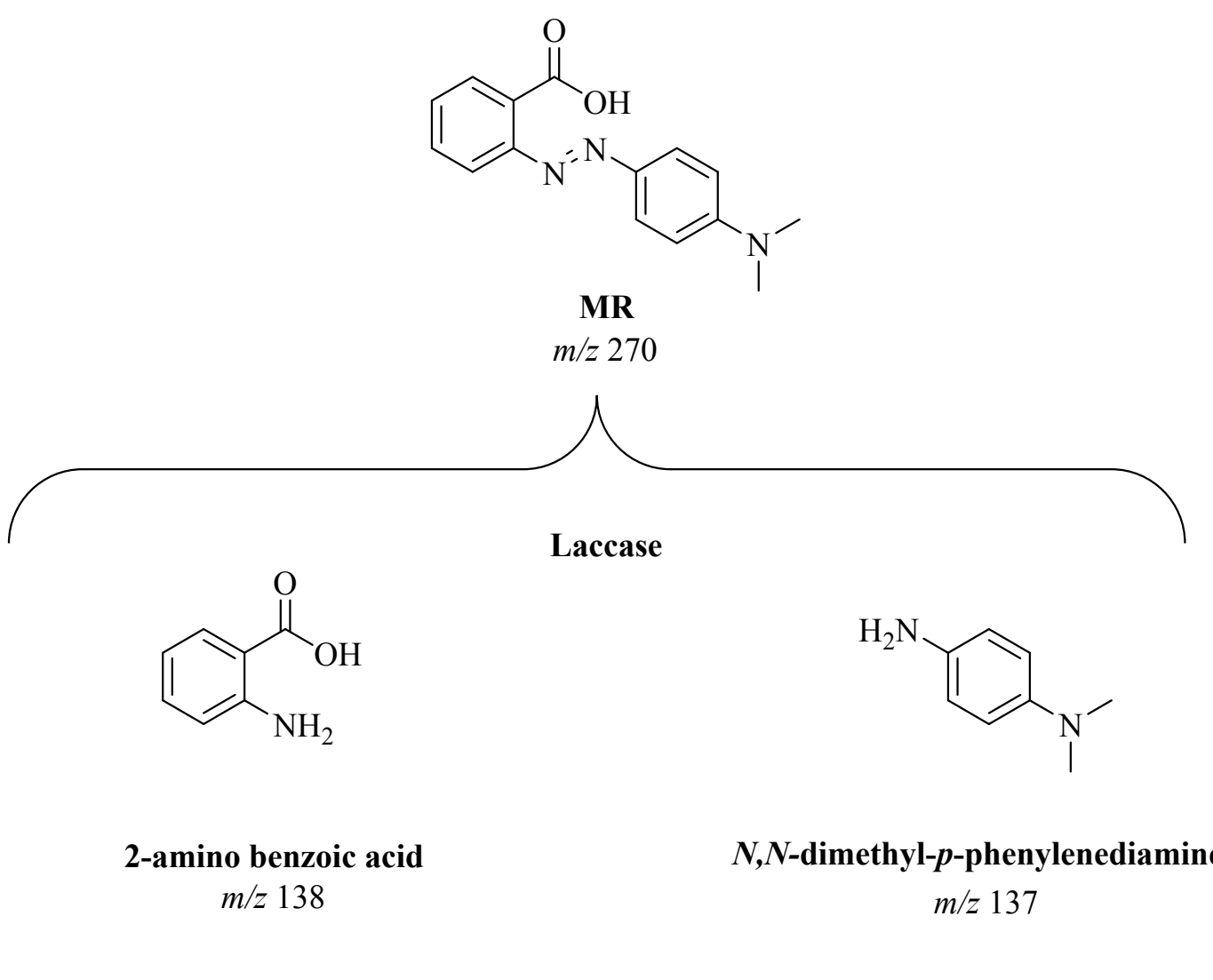

Figure S11. Pathway of methyl red (MR) transformation aided by laccase.[5,6]

ESI-MS analyses of fresh prepared solutions of MB and MR (10^{-5} M in a mixture of 50:50 $H_2$0 and methanol) and the solutions of single MB and MR after 24h decolorization over Laccase-Ny support were acquired with an ion trap mass spectrometer equipped with an ESI ion source (Thermo Scientific LCQ-DECA Ion Max, Thermo Fischer Scientific, San Jose, CA, USA). Samples were acquired in positive ion mode by direct injection into the MS ion source.

Figure S12 reports the mass spectrum of methylene blue and the degradation product resulting from the reaction mediated by the Laccase-Ny system

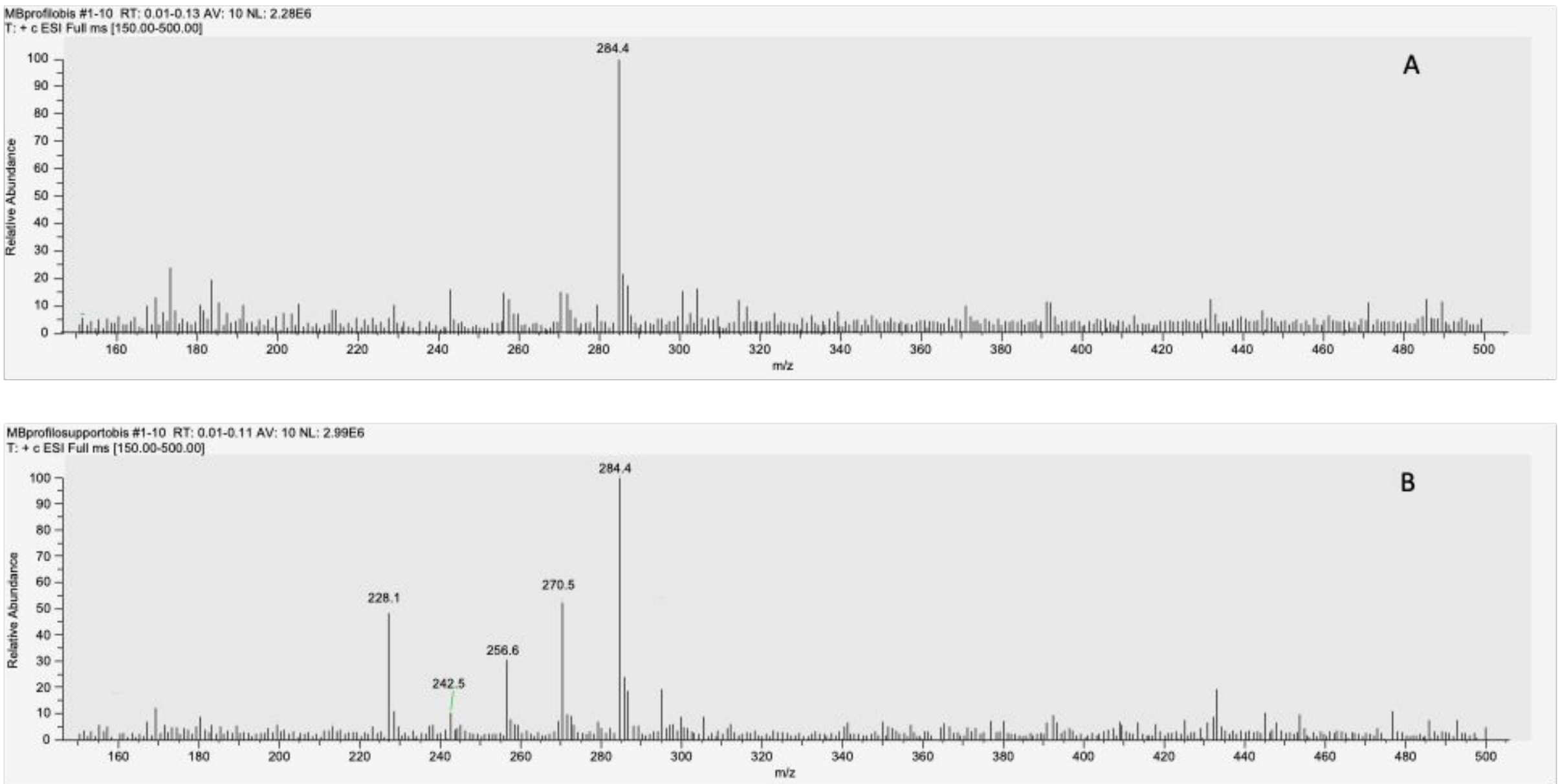

Figure S12. HRMS $[M+H]^+$ spectrum of methylene blue (A) and reaction mixture mediated by the Laccase-Ny system (B).

The MS spectrum of **Figure S13** shows the presence of N-Demethylation products Azure B (*m/z* 270), Azure A (*m/z* 256), and Azure C (*m/z* 242) occurring as the dye's colour progressively fades. Phenothiazine Cleavage (*m/z* 228) is consistent with the central thiazine ring breaking down, forming smaller structures like Thionine.

Conversely, the products of the treatment of MR with Laccase-Ny system are not detectable under the instrumental conditions available to us, likely due to further transformation of the

expected products. However, the spectrum shows a decrease in the percentage abundance of m/z 270 for MR and an increase in background signals (Figure S12).

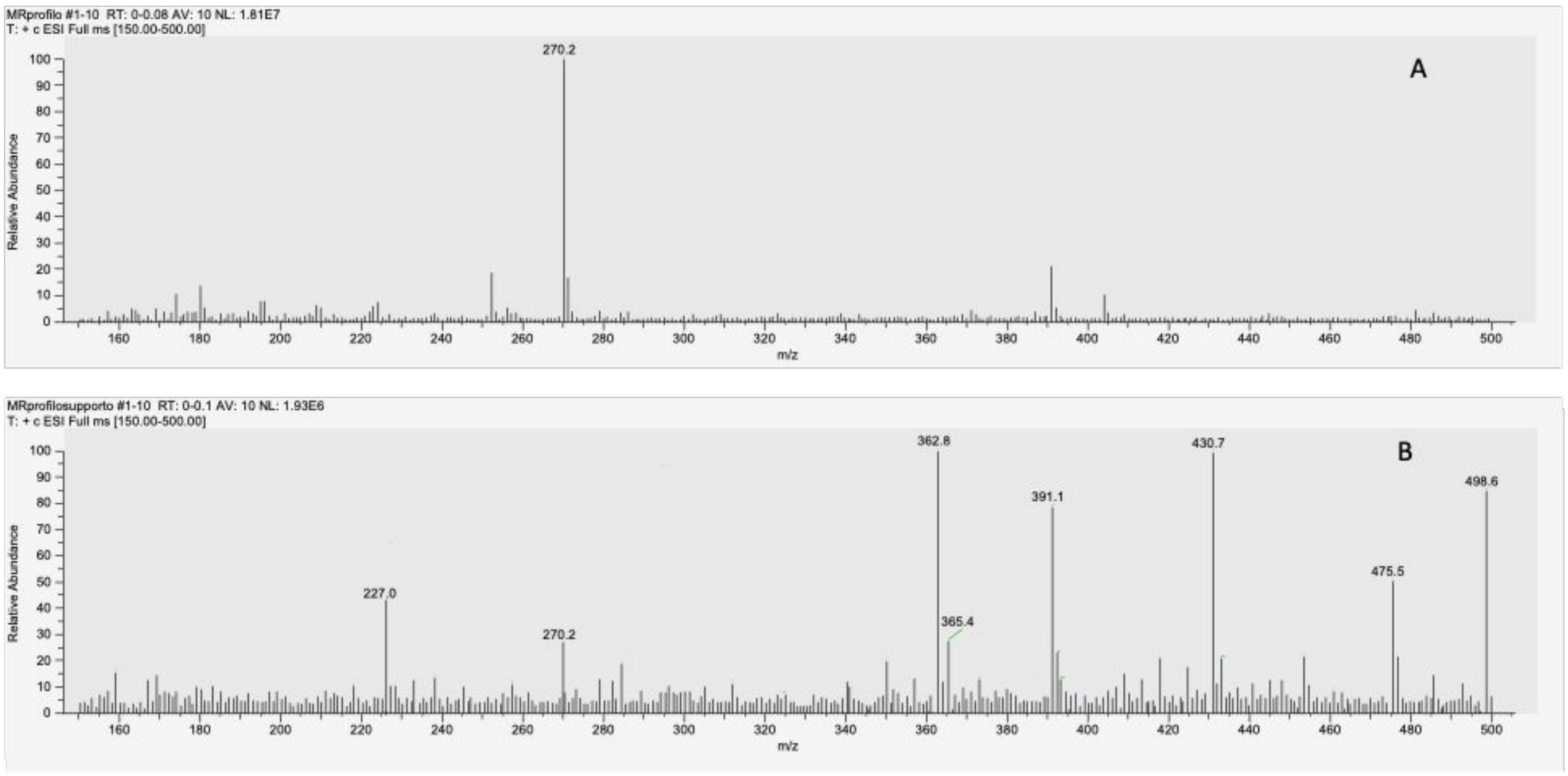

Figure S13. HRMS $[M+H]^+$ spectrum of methyl red (A) and reaction mixture mediated by the Laccase-Ny system (B).

Application of Laccase-Ny system in oxidation reaction

The potential of immobilized LTV support as a recyclable catalyst was evaluated for the oxidation of phenolic compounds, with catechol selected as a model compound. Catechol, a toxic and persistent contaminant frequently discharged from petrochemical, pharmaceutical, and dye manufacturing industries, poses substantial environmental and health risks.[7] We investigated the ability of the newly synthesized system to oxidize the catechol into benzoquinone (**Figure S14**). In detail, Laccase-Ny system (4.4 ± 0.3 mg) was added to a solution of catechol (2.5 mg; 0.023 mmol) in 0.1 M acetate buffer (pH 4.5; 1.5 mL). The reaction was incubated at 25°C and at 200 rpm for 24h. The mixture was partitioned with ethyl acetate (3 × 10 mL). The organic layer was washed with water, dried over anhydrous Na_2SO_4, filtered, and dried under reduced pressure. The conversion of catechol (95.3%) was determined

by HPLC-UV analysis (**Figure S15**), performed using an A 1100 series High-Performance Liquid Chromatography system (Agilent Technologies, Milan, Italy) equipped with a C-18 reversed-phase column (5 µm, 4.60 mm×250 mm, Phenomenex, Castel Maggiore, BO, Italy) kept at 25°C, and with a diode array detector set at 280 nm. The mobile phase consisted of (A) water with 0.1 %(v/v) of formic acid and (B) acetonitrile with 0.1 %(v/v) of formic acid. The solvent gradient was set as follows: 0 – 10 min, 20 – 95 % B; 10 – 15 min, 95 % B; 15 – 25 min, 95 – 20 % B, with 1 mL/min flux. Based on its effectiveness toward catechol, widely recognized as a model phenolic contaminant,[8] the Lacasse-Ny system shows promising potential for further applications in biotechnology, industrial catalysis, and wastewater bioremediation.

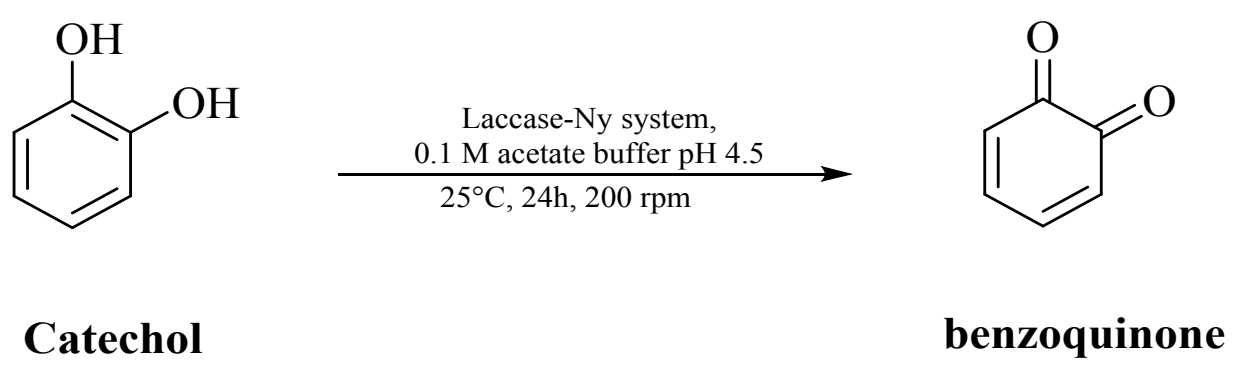

Figure S14. Oxidation reaction of catechol in benzoquinone by Laccase-Ny system.

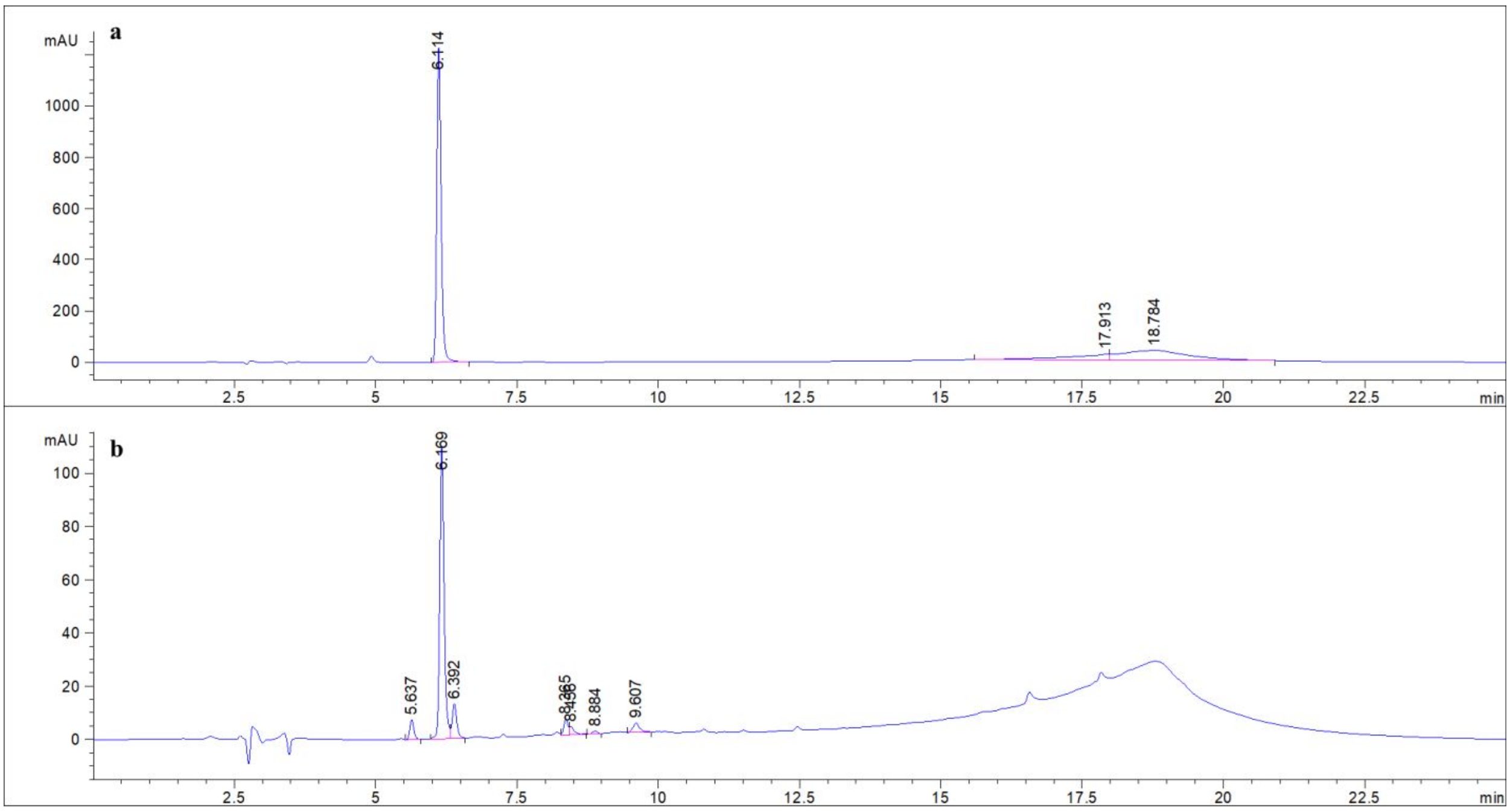

Figure S15. HPLC-UV chromatograms of catechol: before (a) and after the oxidation reaction mediated by Laccase-Ny system (b).

References:

(1) Branchi, B.; Galli, C.; Gentili, P. Kinetics of oxidation of benzyl alcohols by the dication and radical cation of ABTS. Comparison with laccase-ABTS oxidations: an apparent paradox. *Org. Biomol. Chem.* **2005**, *3,* 2604-2614.

(2) Christopher, L.P.; Yao, B.; Ji, Y. Lignin biodegradation with laccase-mediator system. *Systems. Front. Energy Res.* **2014**, *2,* 12.

(3) Sanchez-Alvarez, A.; Quintanilla-Villanueva, G.E.; Rodriguez-Quiroz, O. Rodriguez-Delgado, M.M.; Villarreal-Chiu, J.F.; Sicardi-Segade, A.; Luna-Moreno, D. Use of laccase enzymes as bio-receptors for the organic dye methylene blue in a surface plasmon resonance biosensor. *Sensors.* **2024**, *24,* 8008.

(4) Kangli, W.; Meiling, S.; Xiaomei, P.; Jianqiang, Z.; Zhang, X.; Shen, T.; Tian, Y. Decolourization and biodegradation of methylene blue dye by a ligninolytic enzyme-producing Bacillus thuringiensis: degradation products and pathway. *Enzyme Microb. Technol.* **2022**, *156,* 109999.

(5) Iark, D.; Dos Reis Buzzo, A.J.; Garcia, J.A.A.; Correa, V.G.; Helm, C.V.;Correa, R.C.G.; Peralta, R.A.; Moreira, R.F.P.M.; Bracht, A.; Peralta, R.M. Enzymatic degradation and detoxification of azo dye Congo red by a new laccase from *Oudemansiella canarii. Bioresor. Technol.* **2019***, 289,* 121655.

(6) Takkar, S.; Tyagi, B.; Kumar, N.; Kumari, T.; Iqbal, K.; Varma, A.; Thakur, I.S.; Mishra, A.; Biodegradation of methyl red dye by a novel actinobacterium *Zhihengliuella* sp. ISTPL4: Kinetic studies, isotherm and biodegradation pathway. *Environ. Technol. Innov.* **2022***, 26,* 102348.

(7) Benjelloun, M.; El Messaoudi, N.; Miyah, Y.; Georgin, J.; Franco, D. S. P.; Sehovic, E.; Dehmani, Y.; Knani, S. Recent advances and environmentally friendly methods for catechol removal from wastewater. *JWPE*. **2025**, *76*, 108115.

(8) Monllor-Satoca, D.; Gómez, R. A photoelectrochemical and spectroscopic study of phenol and catechol oxidation on titanium dioxide nanoporous electrodes. *Electrochim. ACTA*. **2010**, 55, 4661-4668.
